# Design and laboratory verification of an AI-driven plant protection robot with a custom communication protocol

**DOI:** 10.1038/s41598-026-49199-3

**Published:** 2026-04-18

**Authors:** Chengzi Jiang, Tianhao Huang, Lijun Zheng, Tianning Xu, Xiaoquan Sun, Heyu Liu

**Affiliations:** 1 Department of Tourism and Marketing, Griffith University, Brisbane, 4111 Australia; 2 Department of Science, Zhijiang College of Zhejiang University of Technology, Shaoxing, 312030 China; 3 Information Engineering College, Zhijiang College of Zhejiang University of Technology, Shaoxing, 312030 China; 4 Hangzhou Sanhua Research Institute Co., Ltd, Hangzhou, 310016 China

**Keywords:** Agricultural robotics, Edge artificial intelligence, Precision spraying, Sensor fusion, Custom communication protocol, Engineering, Mathematics and computing

## Abstract

Traditional agricultural plant protection primarily relies on manual labor and indiscriminate chemical spraying, resulting in inefficiency, increased cost, and environmental pollution. The advancement of precision agriculture is impeded by three persistent bottlenecks in field robotics, namely, unreliable wireless communication within crop canopies, computational limitations for real-time edge artificial intelligence (AI), and high costs of system integration. To address these challenges, as a proof-of-concept feasibility study, this work presents the design and integrated laboratory-based verification of a novel, low-cost, AI-driven plant protection robot. Its core innovation lies in the holistic co-design of a custom CRC-16-protected communication protocol, an edge AI-based pest detection pipeline, and a precision spraying mechanism within a unified architecture. Laboratory-based verification demonstrated that (1) an optimized YOLOv11l model achieves a mean Average Precision (mAP@0.5) of 0.806 for pest detection, with an inference latency of 35.7 ms, on a Raspberry Pi 4B; (2) the custom protocol ensured a data fidelity of 99.91%, with a transmission latency of 12.3 ± 2.1 ms; and (3) the robotic platform achieved a path tracking accuracy of 1.8 ± 0.5 cm and an operational coverage efficiency of 98.7 m²/h, with a projected operational cost of approximately $1.95 per hectare under idealized laboratory conditions. These results confirm the technical feasibility of the integrated approach as a foundation for future field development. This work provides a scalable, cost-effective framework that couples robust perception, reliable communication, and precise actuation, thereby offering a practical proof-of-concept for smart farming applications.

**Supplementary Information:**

The online version contains supplementary material available at 10.1038/s41598-026-49199-3.

## Introduction

Agricultural robots are defined as autonomous equipment designed specifically for agricultural applications and are equipped with advanced capabilities such as precise perception, autonomous decision-making, intelligent control, and automated execution. Their core components generally include a multimodal sensing subsystem, a decision-making and control system, an operational execution mechanism, and an autonomous mobile platform^1–4^. The integration of technologies such as autonomous driving, artificial intelligence (AI), and intelligent control has not only significantly increased the production efficiency but also substantially reduced the labor demand^5,6^.

Among the various types of agricultural robots, plant protection robots represent a prominent category. Such robots are primarily employed for tasks such as weeding, fertilization, pest detection, pesticide application, and crop growth monitoring^7–13^. By combining high-resolution image acquisition, deep learning algorithms, and automated actuation systems, these robots can conduct comprehensive monitoring and provide precise diagnostics of agricultural fields^8,14–18^.

Recent research has significantly advanced the core functionalities needed for autonomous agricultural operations. In the domain of perception and algorithmic development, Paul et al. reported substantial contributions by applying YOLO architectures for crop detection, segmentation, and growth stages classification^19^, by developing models specifically tailored for robust operation in night-time greenhouse environments^20,21^, and by exploring thermal imaging as a supplementary sensing modality^22^. Concurrently, progress has been made in mechanism and system design, including simulation-based dynamic analysis of robotic harvesting carts^23^ and kinematic modeling and AI-optimized design of multi-DOF harvesting robotic arms^24^.

Beyond these perception- and mechanism-focused advances, research has also progressed in the area of related subsystems essential for integrated robotic operation. In the domain of robot environment communication, Sarkar et al.^25^ developed a CoAP-based IoT protocol for mobile robotic systems, achieving a signal reliability of 99% and demonstrating the benefits of lightweight, low-latency protocols for remote control in constrained environments. With respect to multivehicle coordination, Nguyen et al.^26^ proposed a dynamic scheduling framework for multi-AGV systems in which load-aware motion profiling was incorporated, using physically grounded travel time estimation to increase the tracking accuracy and avoid collisions. At the broader system level, He et al.^27^ comprehensively reviewed hand–eye coordination technologies for agricultural robots, covering calibration methods, visual servoing strategies, and their integration in perception action loops, highlighting the critical role of coordinated vision and manipulation in successfully executing complex agronomic tasks. These studies collectively illustrate the rapid progress in individual subsystems that are essential for intelligent agricultural robots.

However, no existing system, whether an academic prototype or a commercial product, provides an integrated, low-cost platform that simultaneously ensures reliable communication, real-time edge AI inference, and precise actuation for plant protection tasks. A significant translational gap remains between advances in subsystem optimization and the deployment of such a fully integrated field robot. Existing implementations often prioritize individual aspects: notably, earlier academic systems, such as the AI-based robot developed by Chen et al., effectively demonstrated targeted applications such as environmental monitoring^28^, whereas contemporary commercial solutions such as Verdant’s SprayBox AI can achieve high precision but at a substantial cost^29^. Consequently, a validated framework that co-designs reliable communication, real-time edge computing, and precise actuation into an affordable, unified platform is lacking. This system-level integration gap is not merely theoretical; this gap is directly manifested as three persistent, interconnected operational challenges in practical, real-time plant protection, namely, (1) unreliable wireless communication within crop canopies (packet loss > 30%^30^, (2) computational bottlenecks for real-time AI on edge devices (latency > 100 ms^31^, and (3) high system integration costs (>$3,000 for commercial solutions^32^.

It is important to clarify that this study represents a proof-of-concept phase in the development cycle of such a complex system. Controlled laboratory environments are intentionally chosen for initial verification to isolate variables, ensure subsystem functionality, and establish a robust technical foundation before advancing to costly and logistically complex field trials. This approach is consistent with established practices in agricultural robotics research, where feasibility is first demonstrated under controlled or simulated conditions^19–27^. By conducting rigorous laboratory-based verification, we aim to confirm the technical feasibility and integrated functionality of the proposed system, thereby providing a necessary foundation for subsequent field-oriented evaluation. Such staged validation is typically considered a critical step to ensure system robustness and reliability prior to real-world deployment.

To bridge the abovementioned gap, a modular, low-cost, and fully integrated AI-driven plant protection robot is proposed in this study. Its core innovation is a system-level co-design approach, in which a custom communication protocol, an edge-deployable AI pipeline, and a precision actuation mechanism are jointly optimized within a unified robotic platform. The principal novelty, therefore, lies not in any standalone component but in this holistic integration, which facilitates the seamless unification of real-time pest detection, adaptive path planning, and targeted spraying through a deterministic data and control loop.

As a proof-of-concept study, the main contributions are as follows:

- A novel, co-designed system architecture for low-cost, AI-driven plant protection that unifies perception, communication, and actuation into a single autonomous robotic platform.
- The design and implementation of a custom, frame-based communication protocol that provides a deterministic and reliable data link essential for real-time coordination between distributed sensors, edge processors, and actuators.
- An edge-computing-based pest detection pipeline in which an optimized deep learning model and a comprehensive training strategy are incorporated, facilitating real-time visual perception within stringent power and latency budgets.
- Mechanical integration and verification of an omnidirectional mobile base with precise motion control and a multi-axis robotic arm, enabling targeted intervention based on the system perception and decision-making.

The remainder of this paper is organized as follows: Sect. 2 details the materials and methods, including the hardware architecture, key mechanism design, and system verification methodology. Section 3 presents and discusses the experimental results. Finally, Sect. 4 concludes the paper and outlines future work.

## Materials and methods

### Overall system hardware architecture

The proposed AI-driven plant protection robot is designed as a unified, mobile platform that entails the integration of distributed environmental perception, edge AI processing, and precise actuation into a single autonomous system. The hardware architecture comprises a suite of coordinated modules, including an STM32 microcontroller, Raspberry Pi and Arduino components, multiple motors, and environmental sensors, which together provide onboard pest detection and targeted intervention.

**Fig. 1:**
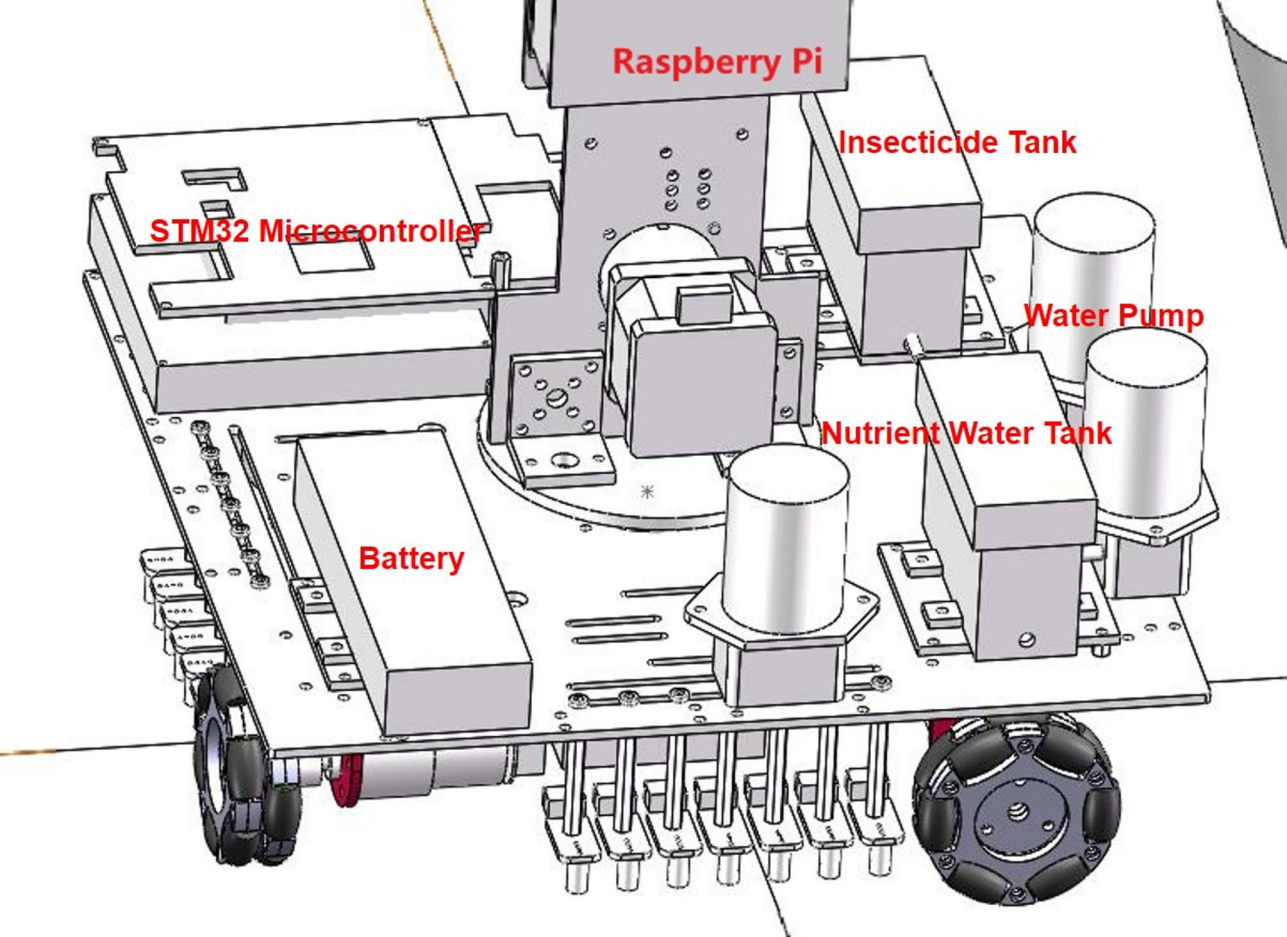
System integration layout showing the spatial arrangement of the core hardware modules.

The spatial arrangement and integration of these core modules on the mobile chassis are shown in Fig. 1. Key components, such as the primary control unit (STM32), the edge AI processor (Raspberry Pi mounted on the robotic arm), and the spraying pumps, are physically integrated to provide the foundation for closed-loop operation. A complete breakdown of all the components, detailing their technical specifications, physical location, primary function, and estimated prototype cost, is provided in Table 1, which serves as the definitive hardware reference.

**Table 1:** Key components, specifications, integration, and functional roles of the proposed plant protection robot.

Component	Technical specifications	Cost (USD)	System location	Primary function
Motion control unit	STM32F407VGT6 microcontroller	~ 17.19	Front-left chassis	Master controller for multi-motor coordination
Omnidirectional wheels	4x DC gear motors, max 0.8 m/s	~ 71.60	Diagonally on chassis	Enables holonomic mobility
Grayscale sensor array	28 channels, 2 cm spacing, 3.3 V	~ 24.06	Under chassis base	Provide feedback for path tracking and calibration
Battery system	Li-ion, 10,000 mAh, 12 V	~ 11.46	Central chassis	Primary power source (~ 4.2 h lab operation)
Water pumps	2x diaphragm pumps	~ 7.16	Main body	Enable targeted spraying of pesticide/nutrient
Three-axis robotic arm	15–35 cm vertical lift, ± 1 mm accuracy	~ 14.32	Main body	Positions end-effector for precise targeting
Stepper motor	42HS3413A4, 1.8°/step	~ 5.73	On arm lift mechanism	Drives vertical positioning via belt-pulley system
Gimbal servo motor	35 kg·cm torque, 360° rotation	~ 5.73	Arm rotation joint	Controls arm’s horizontal rotation
Crank-slider mechanism	*r* = 144 mm, *l* = 155 mm	~ 10.02	On robotic arm	Converts rotary to linear extension
Stretching servo motor	25 kg·cm torque, 180°	~ 5.73	On crank-slider	Drives the crank for extension/retraction
Camera system	External camera for video streams	~ 11.46	Arm end-effector	Captures video stream for pest detection
Display interface	7 inch IPS capacitive touch screen	~ 28.64	On robotic arm	Shows real-time detection results and system status
Edge AI processing unit	Raspberry Pi 4B	~ 100.25	Mounted on arm	Runs YOLOv11l model for real-time pest detection
Spray nozzle	0.4 mm diameter	~ 1.43	Arm end-effector	Delivers targeted spray (3 cm² coverage)
Bluetooth modules	2x HC-05 (10 m)	~ 4.30	Arduino and Raspberry Pi link	Enables data transmission via custom protocol
Data collection terminal	Arduino Mega 2560	~ 4.30	In planting area	Acquires and packetizes environmental sensor data
Environmental sensors	Soil moisture, light, temperature	~ 20.62	In planting area	Monitor key plant growth parameters
Total		~$344.00		

The edge AI processing unit, namely, a Raspberry Pi 4B unit, is physically mounted directly on the base structure of the robotic arm rather than on the main chassis. This placement was a deliberate design choice to minimize the length of the high-bandwidth camera signal cable from the end effector, thereby reducing signal noise and transmission latency. Compared with a chassis-mounted solution, rigid mounting on the arm base also isolates the processor from vibrations of the mobile base more effectively, thereby enhancing operational stability during both navigation and manipulation tasks. Processed commands are then communicated to the main STM32 controller via a short, shielded serial connection, which ensures reliable interprocessor communication.

This integrated architecture constitutes a single, unified robotic system in which the distributed sensor node, edge AI processor, and mobile motion/spraying platform operate cohesively through the custom protocol. This design enables a seamless closed-loop workflow: environmental data are first collected by distributed sensors and packaged by the Arduino unit. The data are then transmitted wirelessly to the core processors of the mobile platform using a custom Bluetooth-based protocol. Following onboard AI processing and decision-making, the system autonomously executes targeted actions, such as precision spraying, through the coordinated control of its omnidirectional drive and three-axis robotic arm.

### Key mechanism design and theoretical modeling

#### Omnidirectional mobility and PID control

**Fig. 2:**
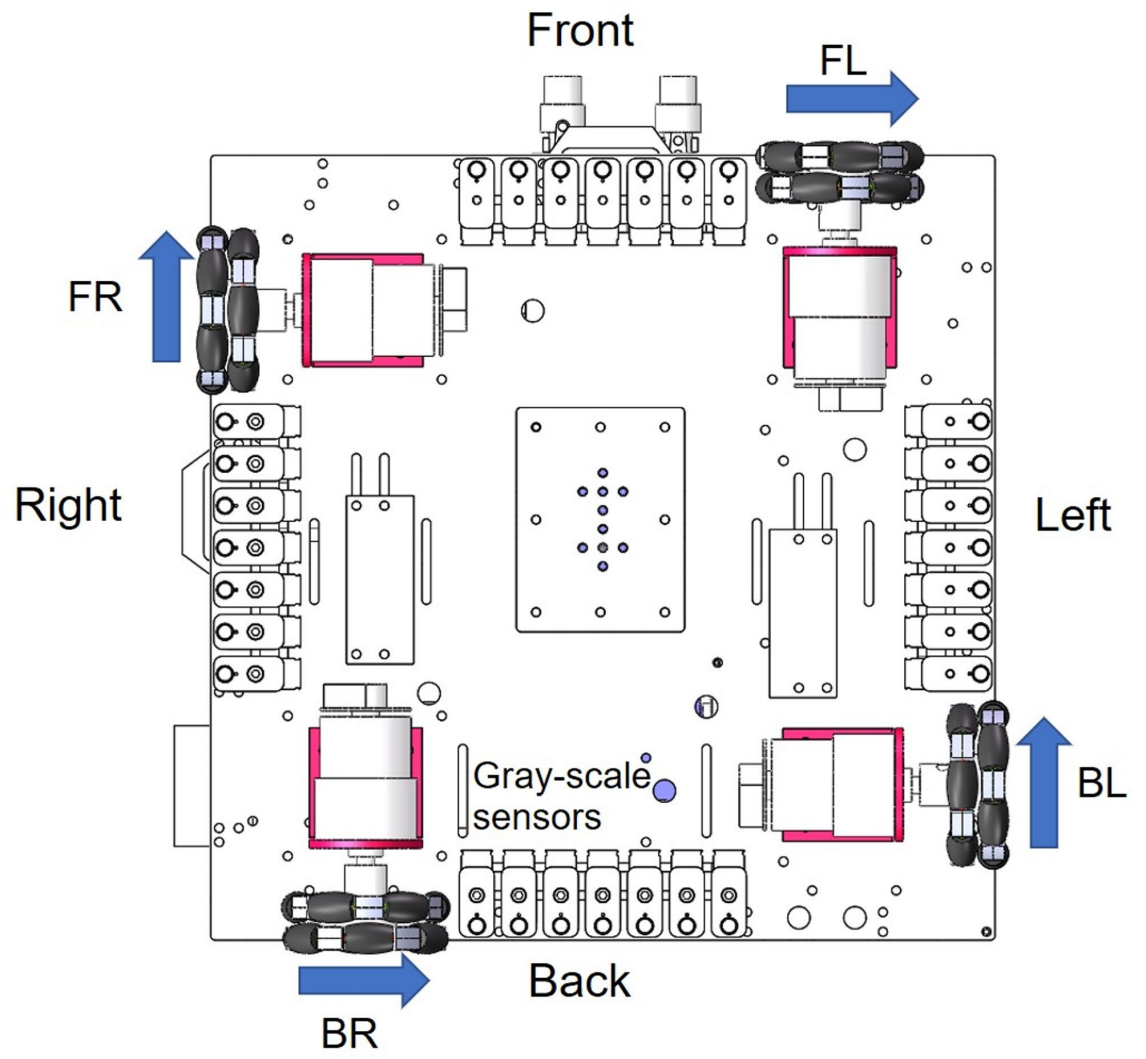
Structural design of the robot base, showing the diagonal configuration of omnidirectional wheels and the grayscale sensor array (view is from the rear looking forward).

To enable holonomic motion, the mobile base of the robot encompasses four Mecanum wheels arranged in a diagonally symmetric pattern, forming the classic “X-type” configuration (Fig. 2). Specifically, the roller axes of the wheel groups at the front-right (FR) and back-left (BL) positions are perpendicular to the forward direction of the robot, primarily providing longitudinal drive. Conversely, the roller axes of the wheel groups at the front-left (FL) and back-right (BR) positions are parallel to the forward direction, which is primarily responsible for providing lateral drive.

With this configuration, planar motion is achieved by coordinating the speed and direction of the four wheel groups. To achieve pure longitudinal translation, the FR and BL wheel groups provide the main driving force, whereas the lateral wheel groups roll freely. Conversely, pure lateral translation is driven by the FL and BR wheel groups. By vectorially synthesizing the velocities of all four wheel groups, the robot can move along any direction within the plane and rotate about its center.

A high-resolution array of 28-channel grayscale sensors (2-cm intersensor spacing) is deployed underneath the chassis base to provide the necessary feedback for closed-loop control. The STM32 microcontroller acquires and preprocesses the data from this sensor array. The embedded control layer then implements a closed-loop operation by continuously comparing the sensed robot position with the target trajectory and computing real-time adjustments to the motor inputs.

The motor control system relies on a discrete-time proportional-integral-derivative (PID) controller to regulate wheel speeds. The control law follows the standard continuous-time form of Eq. 1, which is as follows^33^:

1 $$u(t)={K_p}e(t)+{K_i}\int_{0}^{t} {e(\tau )} \operatorname{d} \tau +{K_d}\frac{{\operatorname{d} e(t)}}{{\operatorname{d} t}}$$

where *u*(*t*) is the computed control output for the motor drivers; *e*(*t*) = *r*(*t*) − *y*(*t*) is the instantaneous tracking error, with *r*(*t*) denoting the desired trajectory from the S-shaped planner and *y*(*t*) denoting the actual robot position derived from the 28-channel grayscale sensor array; and *K*_*p*​_, *K*_*i*​_, and *K*_*d*_​ denote the tuned proportional, integral, and derivative gains, respectively, for the mobile base.

The PID gains were tuned via a combined model-based and empirical approach. Initial gain values were simulated with a simplified kinematic model of the Mecanum wheel platform in MATLAB/Simulink. These initial values were then fine-tuned for the physical prototype through the Ziegler−Nichols step response method, followed by manual optimization to achieve the desired tracking performance. Sensitivity analysis revealed that the path tracking accuracy of the system is most sensitive to *K*_*p*_. A deviation greater than ± 15% from the experimentally determined optimal *K*_*p*_ value of 0.8 resulted in either oscillatory behavior or a steady-state error exceeding the acceptable ± 2 cm limit value. Notably, *K*_*i*_ was set to a small value of 1.2 primarily to eliminate minor steady-state offsets caused by uneven wheel friction, without introducing significant integral windup during turning maneuvers. Moreover, *K*_*d*_ was set to 0.05 to provide moderate damping without amplifying sensor noise.

#### Robotic arm kinematic modeling

**Fig. 3:**
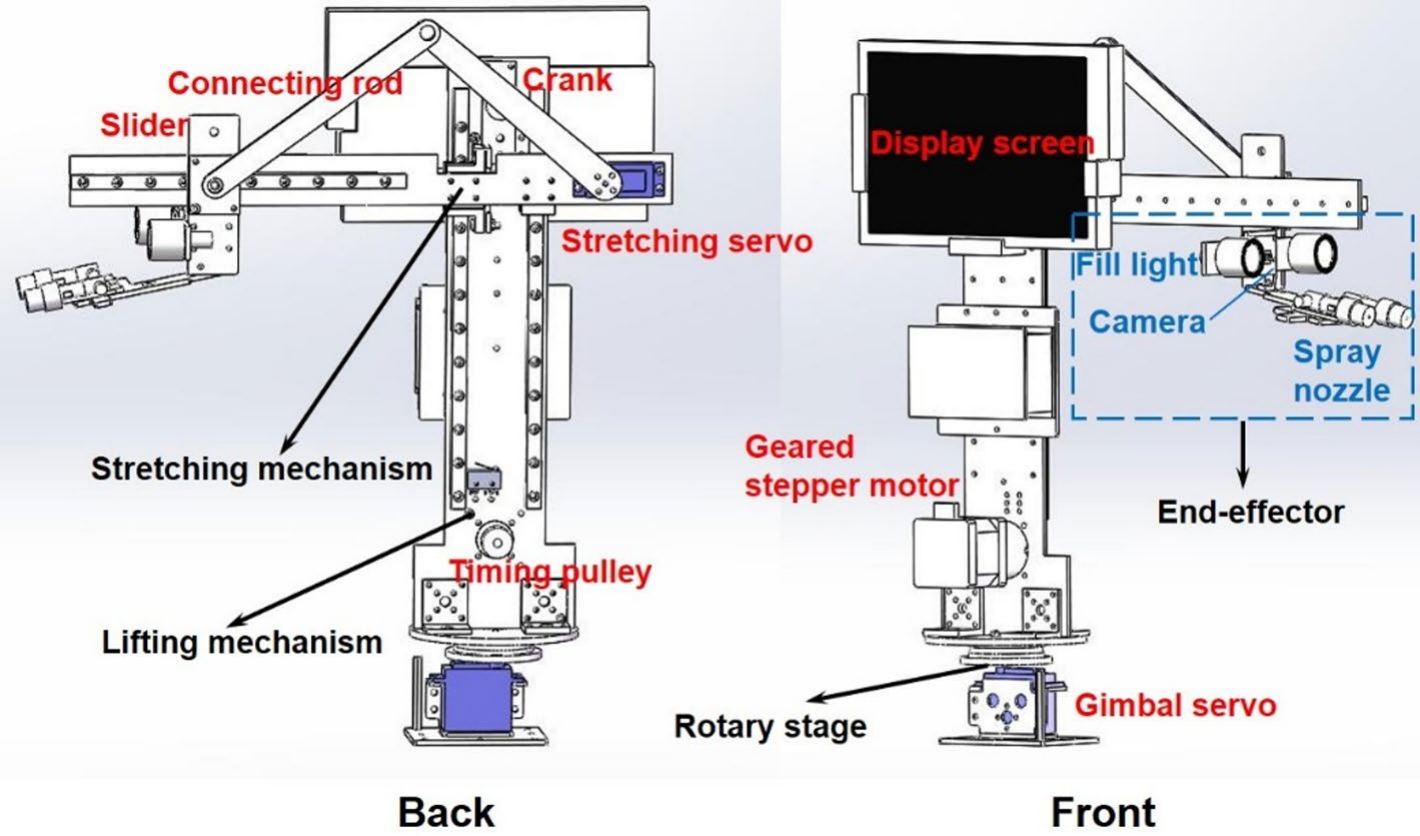
Three-axis robotic arm design featuring belt−pulley lifting and crank−slider stretching mechanisms for precise end-effector positioning.

To achieve precise positioning of target plants, the system employs a three-degree-of-freedom robotic arm, as shown in Fig. 3. The arm comprises three coordinated mechanisms, namely, a rotary mechanism, a lifting mechanism, and a stretching mechanism, ultimately yielding a multifunctional end effector. The rotary mechanism, located at the base and driven by a servo motor, provides 360° horizontal rotation for the entire arm. Mounted above, the lifting mechanism is driven by a geared stepper motor through a timing-belt system, moving the upper assembly smoothly along vertical linear guides to adjust the end-effector height. The stretching mechanism, mounted on the lifting mechanism, is based on the crank−slider principle and actuated by another servo motor, thereby enabling precise extension and retraction of the end effector along horizontal guides. A camera, fill lights, and a spray nozzle are integrated at the end effector. By coordinating the motions of these three degrees of freedom, the robotic arm can rapidly and accurately position the end effector at target points in three-dimensional space, thereby providing the essential hardware support for a vision-based precision spraying system.

The lifting mechanism provides a vertical travel range of 15 to 35 cm. Movement is driven primarily by the frictional force generated from belt tension in the pulley system, which transmits motion to the driven wheel. A custom clamping device secures the belt, allowing the robotic arm assembly to ascend and descend along linear guides (slider and slide rail). An integrated tensioning mechanism is used to adjust the belt−pulley contact angle to prevent belt slippage. Dual limit switches define the operational boundaries. Displacement control of the lifting mechanism is achieved via a stepper motor. The required pulse count *N* can be calculated by Eq. 2 based on the target displacement^34^:

2 $$N=\frac{n}{s}d$$

where *d* is the target vertical displacement (e.g., 20 mm) of the robotic arm assembly, *n* is the step resolution of the motor (1600 pulses/revolution), and *s* is the linear displacement per motor revolution (5 mm/rev) determined by the belt−pulley system in our design.

The stretching mechanism relies on a crank−slider design that converts rotational motion of the crank into linear motion of the slider, enabling smooth extension and retraction along the rail. Based on the geometric constraints of the crank−slider mechanism, the horizontal displacement *x* of the slider is given by Eq. 3^35^:

3 $$x(t)=r\cos \theta +\sqrt {{l^2} - {r^2}{{\sin }^2}\theta }$$

The corresponding velocity *v*(*t*) and acceleration *a*(*t*) can be obtained as the first and second time derivatives of *x*(*t*) (Eqs. 4 and 5), respectively.

4 $$v(t)= - \omega r\sin \theta - \frac{{\omega {r^2}\sin 2\theta }}{{2\sqrt {{l^2} - {r^2}{{\sin }^2}\theta } }}$$

5 $$a(t)= - {\omega ^2}r\cos \theta - \frac{{{\omega ^2}{r^2}({l^2}\cos 2\theta +{r^2}{{\sin }^4}\theta )}}{{{{({l^2} - {r^2}{{\sin }^2}\theta )}^{{3 \mathord{\left/ {\vphantom {3 2}} \right. \kern-0pt} 2}}}}}$$

where *θ* = *ωt* is the crank angle; *ω* is the constant angular velocity (10 rad/s in our design); and *r* and *l* denote the crank and connecting rod lengths (144 and 155 mm, respectively, in our design).

### Path planning algorithm design

The path planning strategy for the plant protection robot is designed to ensure complete, gap-free coverage of rectangular crop rows, in compliance with horticultural spraying protocols. The design aims to reduce the total travel distance, operation time, and number of energy-intensive turns.

**Fig. 4:**
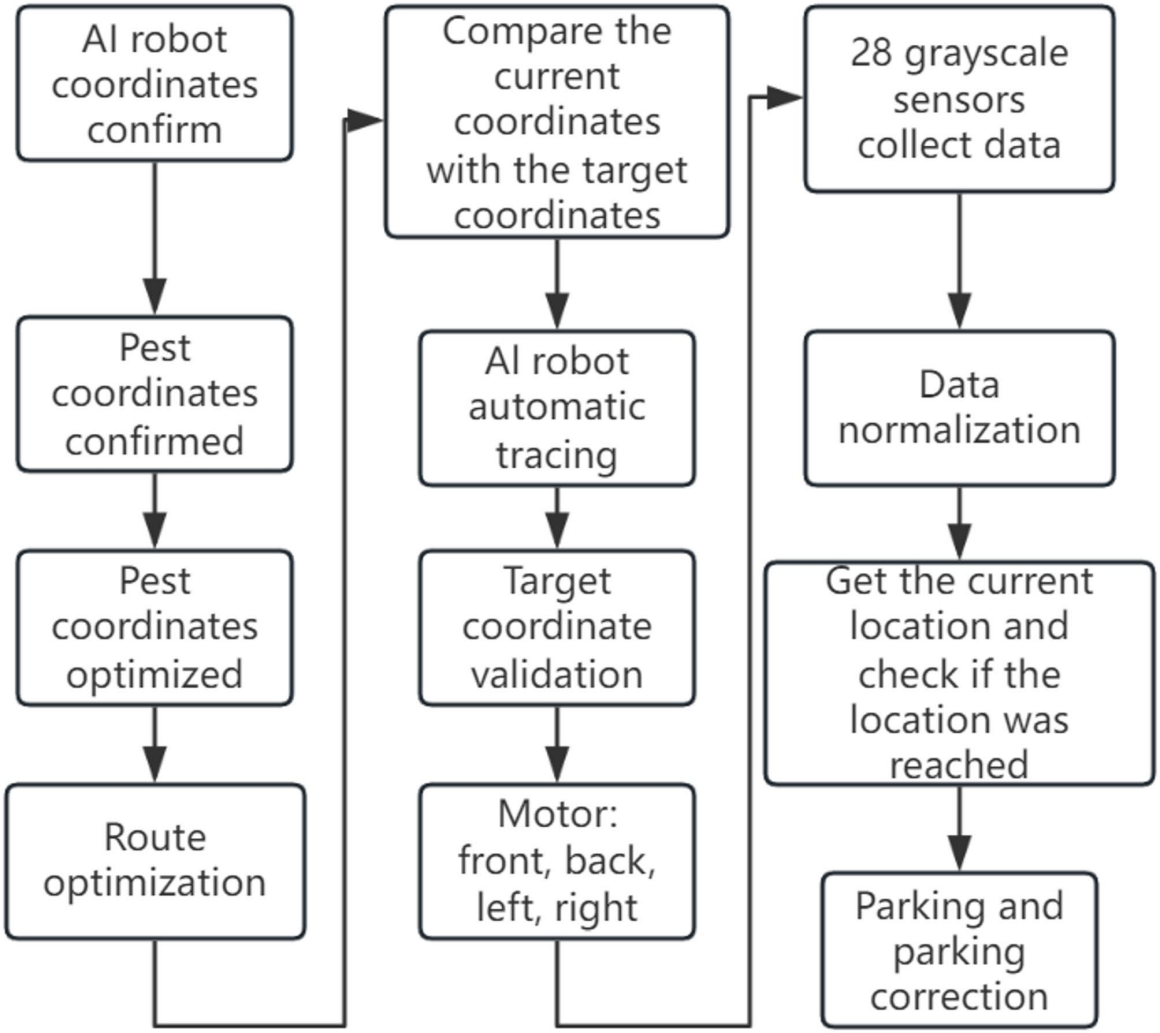
Workflow of the S-shaped coverage path planning algorithm for uniform spraying.

The system relies on a predefined field coordinate system for localization. Based on its position, the robot generates an S-shaped coverage path optimized for efficient traversal along the rows. The workflow of this planning process is shown in Fig. 4.

The optimization objective for generating the S-shaped coverage path is to minimize the total travel distance. Given the rectilinear layout of crop rows, this objective can be formulated using the Manhattan distance^36^:

6 $$\mathop {\hbox{min} }\limits_{P} \sum\limits_{{k=1}}^{{K - 1}} {(\left| {{x_{k+1}} - {x_k}} \right|+\left| {{y_{k+1}} - {y_k}} \right|)}$$

subject to **p**_*k*​_ ∈ *C*_free_ ​∀*k* = 1, 2, …, *K*.

where *P* = {**p**_1_​, **p**_2_​, …, **p**_*K*_​} denotes the sequence of robot waypoints in the field coordinate system; **p**_*k* ​_= (*x*_*k*​_, *y*_*k*​_) denotes the 2D coordinates of the center of the mobile base at the *k*-th waypoint; *C*_free_​ denotes the obstacle-free region defined by the interrow spacing; and the objective function yields the Manhattan distance, which accurately reflects the rectilinear movement of the robot along crop rows and across alleys.

**Fig. 5:**
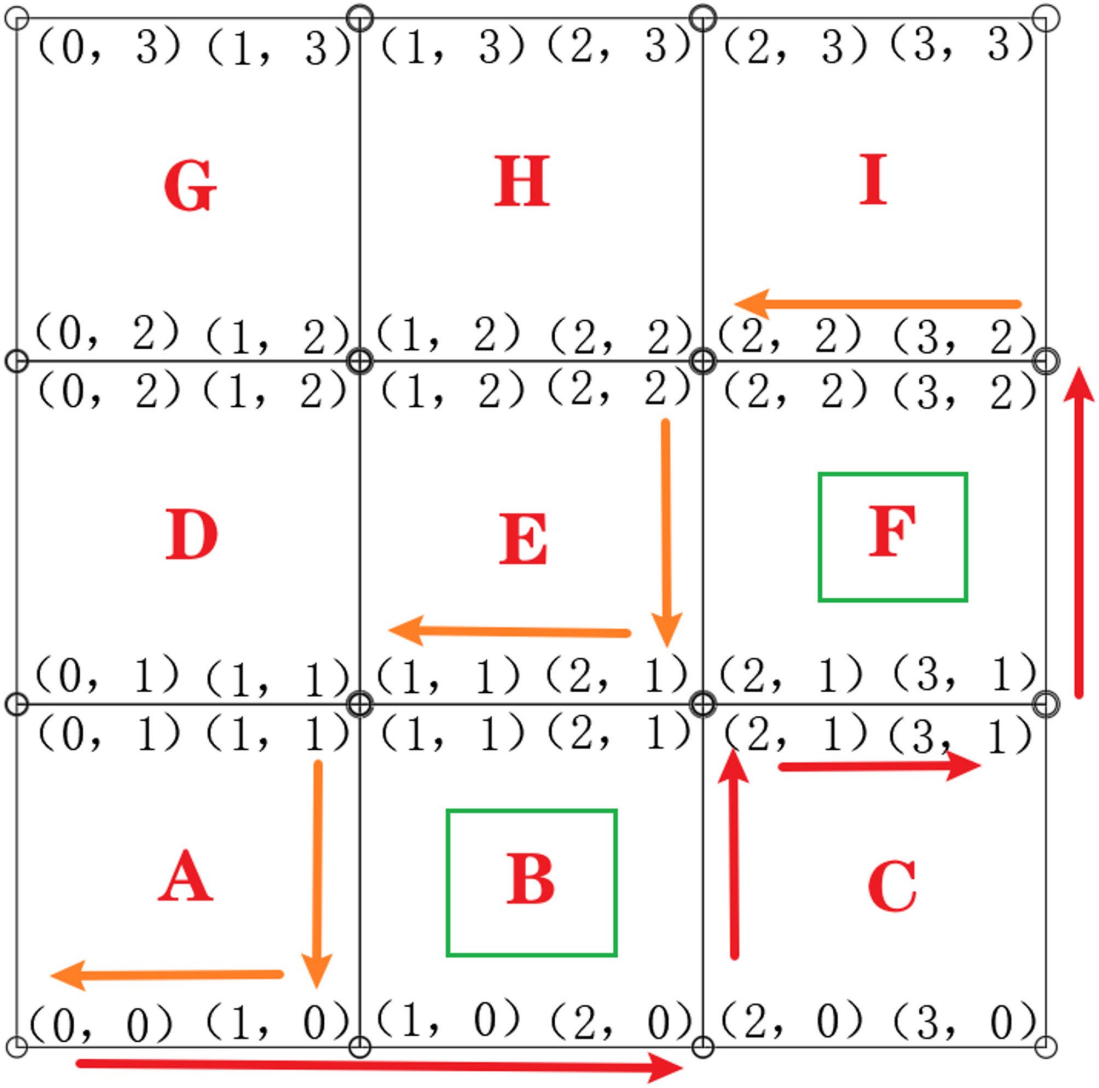
Example of the path optimization algorithm using diagonal coordinate mapping. The planned route from (0,0) to (3,2) via shared waypoint (2,1) demonstrates efficient traversal between infected areas B and F with minimal redundant turns.

The optimization process governed by Eq. 6, which is based on a coordinate mapping rule, is conceptually shown in Fig. 5 using a 3 × 3 grid for clarity. In this representation, each planting area is assigned coordinates. When areas require treatment (e.g., areas B and F in Fig. 5), the algorithm identifies shared and diagonal coordinates as critical waypoints to design an efficient round-trip path that minimizes redundant turns. For instance, the planned route from (0,0) to (3,2) via (2,1) demonstrates how the algorithm arranges waypoints to achieve comprehensive coverage. The path segments in this example are strictly axial (horizontal or vertical), rendering the Manhattan distance an exact measure of the actual travel distance. This coordinate-based rule and S-shaped pattern generation are generalizable, with the grid scale determined by the actual dimensions and resolution of the target field.

### Edge AI-based pest detection pipeline

#### Model selection and dataset

Building upon the established success of the YOLO architecture in agricultural vision tasks, such as crop detection, segmentation, and growth stage determination in controlled environments^19–22^, the state-of-the-art YOLOv11l object detector^37^ is adopted herein for pest detection. This model was selected because of its favorable balance between high accuracy and high inference speed, which is a critical requirement for real-time deployment on edge-computing robotic platforms. The YOLOv11l variant contains 631 layers with 25.3 million parameters and a computational load of 86.6 GFLOPs. Its architecture entails the incorporation of advanced modules such as C3k2 for efficient feature extraction, C2PSA for spatial attention, and SPPF for multiscale feature fusion, making it well suited for detecting variably sized pests in complex environments.

The selection of the YOLOv11l variant over lighter or heavier alternatives was informed by systematic benchmarking on the target edge platform, a Raspberry Pi 4B unit. Lighter variants such as YOLOv11n and YOLOv11s could offer inference speeds exceeding 40 FPS, but preliminary testing revealed that they suffer a 10 − 15% reduction in mAP for the pest dataset, which could lead to unacceptable missed detections in practical applications. Heavier variants such as YOLOv11x, while potentially offering higher accuracy, exceeded the available memory bandwidth of the Raspberry Pi 4B unit, causing unstable inference and frequent frame drops. The YOLOv11l variant, with 25.3 million parameters and 86.6 GFLOPs, provided the optimal trade-off, achieving a real-time throughput of 28 FPS while maintaining the high accuracy needed for reliable pest detection, as supported by the results presented in Sect. 3.3.

 A custom dataset of 720 high-resolution images (640 × 640 pixels) was compiled for model training and evaluation. The dataset covers nine distinct pest classes common in agricultural settings, as detailed in Table S1. Each image was meticulously annotated with bounding boxes in YOLO format. To ensure robust evaluation of the generalization performance and to prevent data leakage, the dataset was partitioned into a training set of 600 images (83.3%) and a validation set of 120 images (16.7%), as summarized in Table 2.

**Table 2:** Pest detection dataset summary.

Attribute	Details
Total images	720
Training set	600 (83.3%)
Validation set	120 (16.7%)
Number of classes	9
Image resolution	640 × 640
Annotation format	YOLO format (normalized coordinates)

 To significantly enhance model robustness under realistic conditions of varying lighting, occlusion, and orientation levels, an extensive data augmentation pipeline was applied during training. The pipeline included Mosaic augmentation, random horizontal flipping, spatial transformations (translation and scaling), and comprehensive color adjustments in Hue, Saturation, Value (HSV) space, with specific parameters detailed in Table S2.

#### Training and optimization configuration

 The YOLOv11l model was trained from scratch for 300 epochs with a batch size of 32. Training was performed using the AdamW optimizer^38^ with an initial learning rate of 0.01, a momentum of 0.937, and an L2 weight decay of 0.0005, as detailed in Table S3. To stabilize the early training phase, a linear warm-up schedule was applied over the first 3 epochs, followed by a cosine annealing schedule for ensuring controlled learning-rate decay.

The overall training objective was to minimize a multitask loss function *L*_total_ defined as Eq. 7^39^:

7 $${L_{{\mathrm{total}}}}={\lambda _{{\mathrm{box}}}} \cdot {L_{{\mathrm{box}}}}~+{\lambda _{{\mathrm{cls}}}} \cdot {L_{{\mathrm{cls}}}}+{\lambda _{{\mathrm{dfl}}}} \cdot {L_{{\mathrm{dfl}}}}$$

where *L*_box_, *L*_cls_, and *L*_dfl_ represent the bounding box regression loss, classification loss, and Distribution Focal Loss (DFL)^40^, respectively. The corresponding loss weights were defined as *λ*_box_ = 7.5, *λ*_cls_ = 0.5, and *λ*_dfl_ = 1.5 (Table S4), respectively, prioritizing localization accuracy for the robotic application.

To increase the generalizability of the model, the comprehensive data augmentation pipeline described in Sect. 2.4.1 (parameters in Table S2) was applied throughout training. During inference, non-maximum suppression with an IoU threshold of 0.7 was used to eliminate redundant detections.

### Custom communication protocol design

**Fig. 6:**
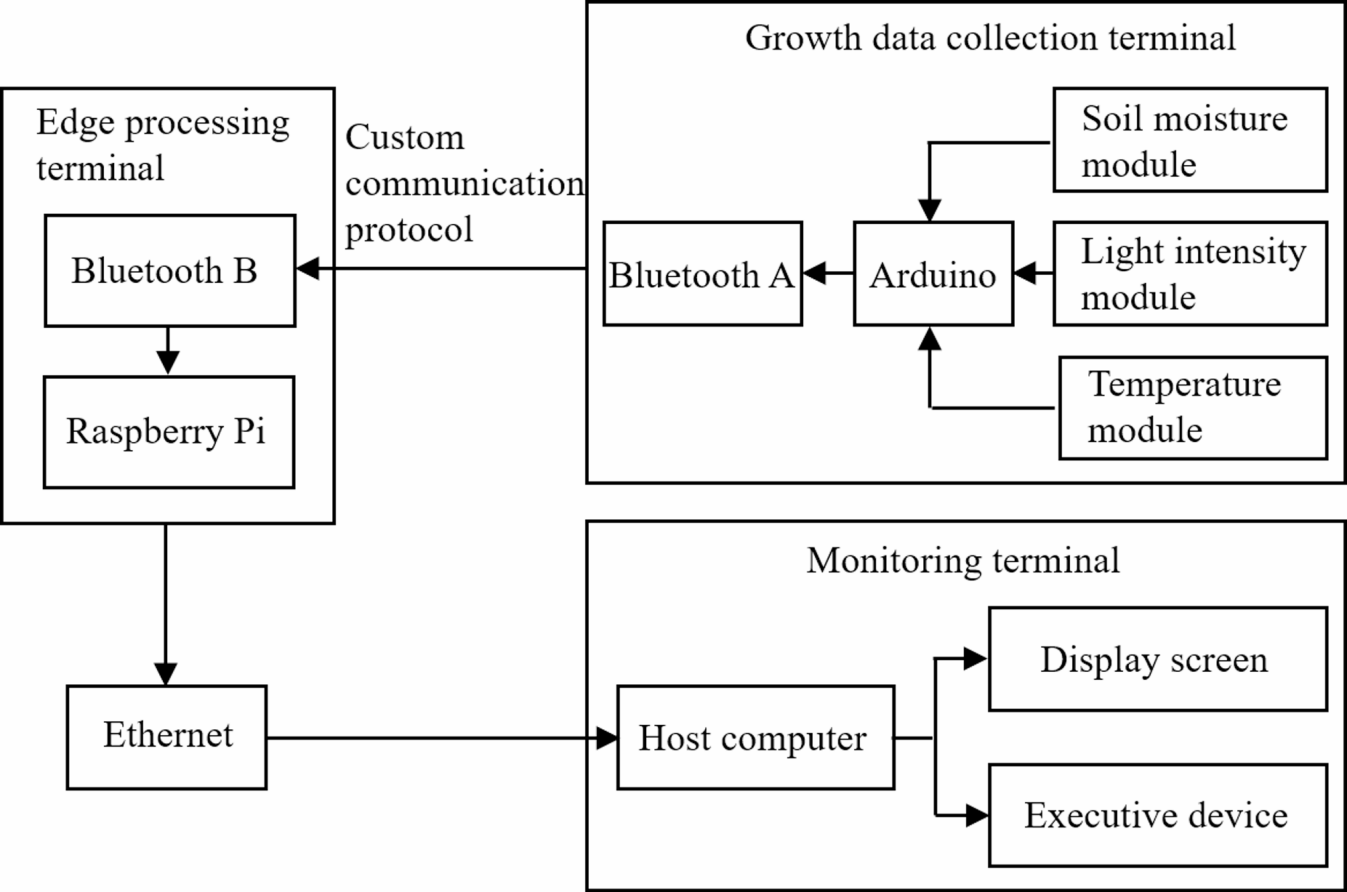
Architecture of the plant growth monitoring and interaction system based on custom protocol-based data flow.

The custom communication protocol is central to the plant growth monitoring and interaction system shown in Fig. 6. In this architecture, a distributed data collection terminal (Arduino Mega 2560) acquires key environmental parameters (soil moisture, light intensity, and temperature) and wirelessly transmits the data to the mobile edge-processing unit (Raspberry Pi) via Bluetooth using this protocol. Upon reception and decoding, the data are assessed against optimal growth thresholds to enable responsive control.

The protocol design was driven by two primary considerations. First, real-time control requires low latency, deterministic timing, and minimal software overhead. Although protocols such as ZigBee and LoRa excel in long-range, low-power sensor networks^41^, they are optimized for periodic data collection rather than millisecond-level control. Similarly, MQTT relies on a stable network infrastructure and can introduce unpredictable latency under dynamic conditions^42^. Second, the tight integration among sensing, AI, and actuation requires a simple, point-to-point data link. While the Robot Operating System (ROS) is a standard for complex robotics, its middleware imposes an overhead that is prohibitive for microcontrollers such as the STM32^43^.

To satisfy these requirements, a lightweight, frame-based custom protocol was developed. Its novelty lies not in the standard CRC-16 check, but in its holistic co-design with the overall system architecture. Functioning as a dedicated nervous system, it connects the Arduino-based sensor node, the Raspberry Pi AI engine, and the STM32 motion controller through a minimal, byte-efficient format, thus establishing a deterministic data link. This integrated approach eliminates unnecessary protocol negotiation and header overhead. Combined with a simple frame structure, this design ensures that time-critical commands (e.g., for spray actuation or emergency stop) can be encoded, transmitted, and decoded with a deterministic latency smaller than 20 ms in our laboratory setup, which is a key requirement for closed-loop robotic response.

**Fig. 7:**
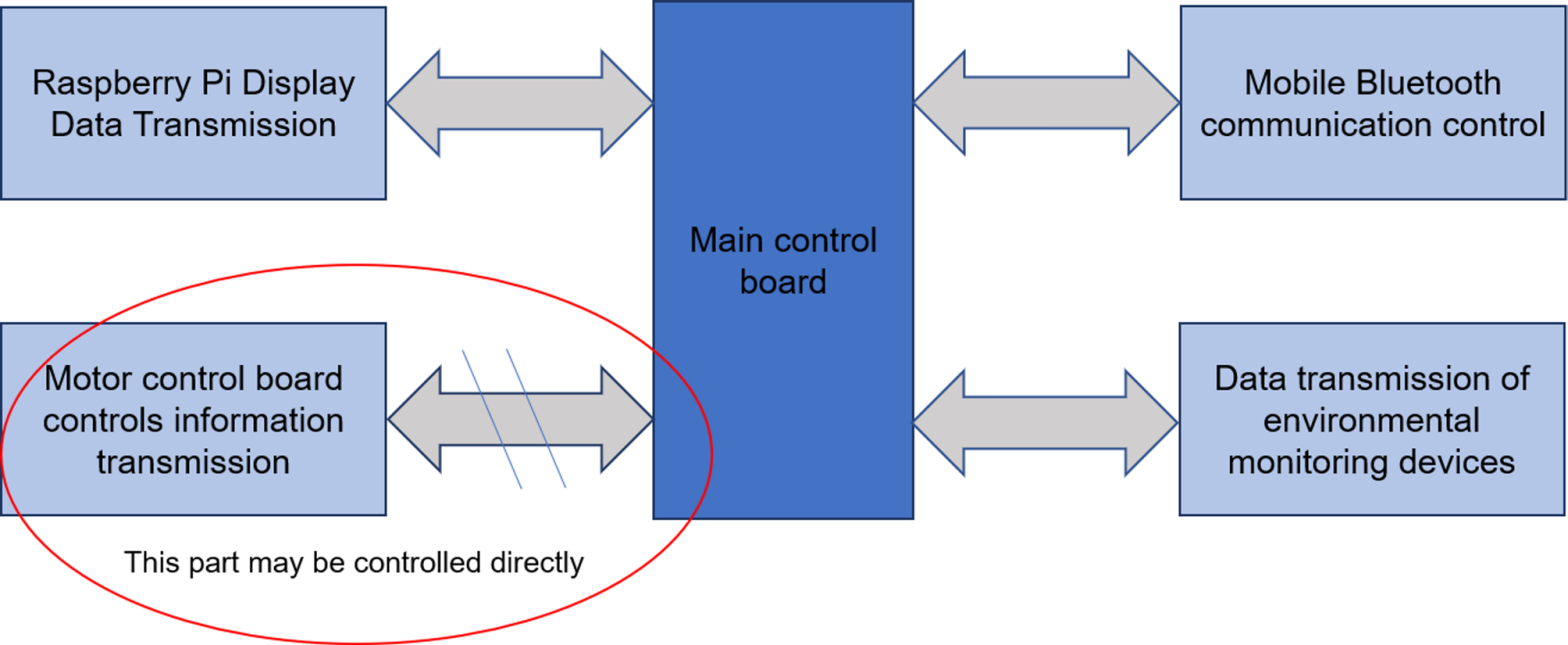
Frame structure of the custom communication protocol with CRC-16 protection.

**Fig. 8:**
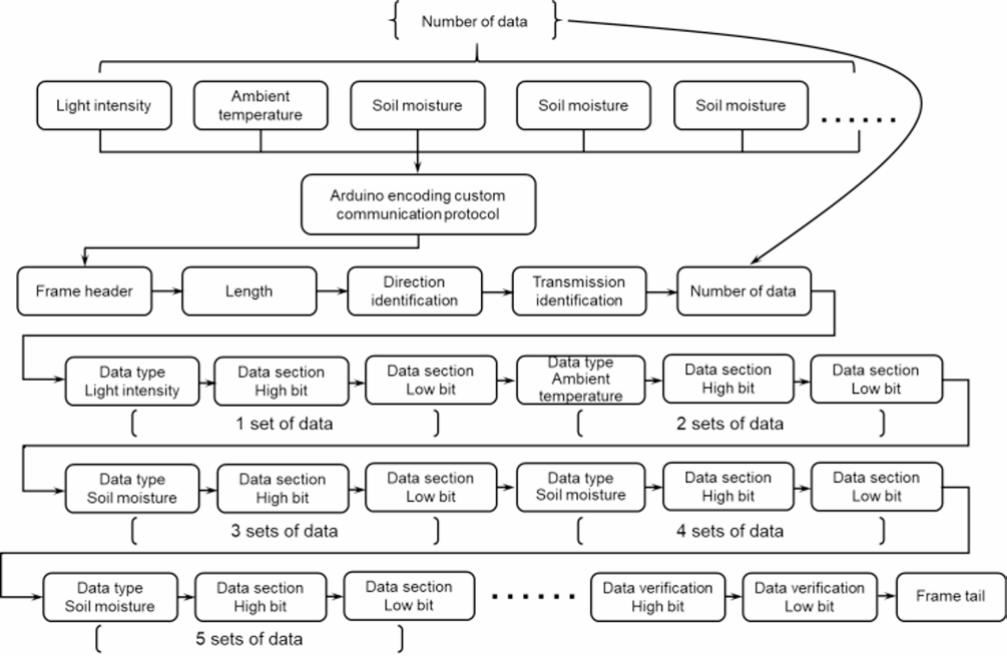
Protocol encoding process for packaging sensor data into transmission frames.

 To ensure reliable transmission, the protocol relies on the frame structure shown in Figs. 7 and 8. Each frame is bounded by a fixed Start Character (0x68) and End Character (0x16). Among these delimiters, several control fields govern data flow, including a Length Field (0x00 − 0xFF) encoding the total frame size excluding the delimiters and a Direction Identifier specifying the routing (e.g., 0x12 for sensor-to-edge uplink and 0x02 for command downlink). The core Payload carries the operational data and is structured as follows: a 1-byte Transmission Identifier (0x00 for sensor/text data according to Table 3; 0x11 for control commands according to Table 4), followed by a Data Count byte (0x00 − 0x0F, indicating the number of active sensor modules, ≤ 15). Subsequent entries for each sensor module comprise a Data Type byte (e.g., 0x01 for light; 0x02 for moisture) paired with a corresponding 2-byte Data Section (raw values from 0x0000 to 0xFFFF).

**Table 3:** Categorization of text data.

Data count: 1 byte (number of data entries, maximum 3)	
Data type: 1 byte	Data section: 2 bytes (integer values only)
Light intensity 000000001	Two bytes combined as u16; unit depends on data type
Soil moisture 000000010	
Ambient temperature 000000011	
Reserved	

**Table 4:** Categorization of control data.

Data type: 1 byte	Data section: 2 bytes (integer values only)
Moving coordinates 00000001	First byte: X coordinate; last byte: Y coordinate
Bottom servo control 00000010	Two bytes combined as u16; value represents angle in °
Stretching servo 00000011	Two bytes combined as u16; value represents angle in °
Height control stepper motor 00000100	Two bytes combined as u16; value represents height in mm
Water pump 1 control 00000101	Two bytes combined as u16; value represents injection time in ms
Water pump 2 control 00000110	Two bytes combined as u16; value represents injection time in ms
Fill-in light control 00000111	First byte: 0 (reserved); last byte: 0 = off, 1 = on
Raspberry Pi camera control 00001000	First byte: 0 (reserved); last byte: 0 = off, 1 = on
Automatic control 00001001	First byte: 0 (reserved); last byte: boot mode (0 − 255)

The Frame Check Sequence (FCS) provides integrity verification via a CRC-16 algorithm using the CCITT polynomial (*x*^16^ + *x*^12^ + *x*^5^ + 1)^44^:

- Initialize the register: *R* ← 0xFFFF.
- For each byte *B*_*i*​_ in the data field:
  - *R* ← *R* ⊕ *B*_*i*_ (bitwise XOR).
  - Perform 8 iterations on *R*:
    If the most significant bit (MSB) of *R* is 1, *R* ← (*R* ≪ 1) ⊕ 0x1021.
    Otherwise, *R* ← *R* ≪ 1.
- The final value of *R* is the FCS.

This algorithm detects all single-bit, double-bit, and odd-numbered errors, as well as burst errors up to 16 bits in length, with guaranteed effectiveness for a bit error rate (BER) < 10^− 5^.

During operation, the Arduino model is established and transmits frames. The Raspberry Pi unit verifies each received frame through a multistage process that includes recomputing the FCS. Valid frames are parsed for further processing, whereas corrupted frames are discarded.

### System verification methodology

This study represents a proof-of-concept phase in the development of an integrated agricultural robot. Accordingly, all verification activities were intentionally conducted under controlled laboratory conditions. This controlled approach facilitates the isolation of variables, ensures repeatable testing of individual subsystems, and establishes baseline performance benchmarks prior to more complex and resource-intensive field trials. The following subsections detail the methods employed for virtual design verification, functional testing of the physical prototype, and quantitative performance evaluation for each core subsystem.

#### Verification of the virtual design with SOLIDWORKS

All mechanical components of the robot were modeled and virtually assembled in SOLIDWORKS 2019 (Figs. 1, 2 and 3). Static interference analyses were conducted across the designed range of motion, including robotic arm lifting and slider extension, via the software’s Interference Check function. This virtual verification confirmed the absence of partial collisions under ideal kinematic assumptions, thus establishing the foundational feasibility of the mechanical design for subsequent physical prototyping. Note that this step constitutes design verification rather than dynamic performance validation.

#### Laboratory-based functional verification

To verify the physical realizability and basic functional integration of the design, a series of functional verification tests were conducted for the assembled robot prototype under controlled laboratory conditions. All tests were performed on a flat, uniform concrete surface to minimize interference from terrain variations, with a fully charged battery (12 V; 10,000 mAh) providing consistent power.

Subsystem tests confirmed the expected functionalities. The omnidirectional mobility was verified by issuing predefined velocity commands, such as forward, lateral, and rotational motions, and visually observing the corresponding chassis movements. The robotic arm lift mechanism traversed its full designed range of 15–35 cm, while the stretching mechanism operated smoothly. The camera successfully captured live images. The data acquisition and communication link was verified by placing the sensor node at a fixed distance of 5 m, collecting a sample dataset, and confirming its successful reception and decoding in the monitoring interface of Raspberry Pi. Both water pumps could be independently activated for spraying.

Finally, an integrated workflow test was executed. A single high-level command sequence was issued, which included navigation to a marked coordinate, positioning of the robotic arm, and triggering of a brief spray pulse. The prototype successfully completed this automated sequence, confirming the fundamental electrical connectivity, logical interoperability, and correct sequencing of all hardware modules.

#### Quantitative performance evaluation and statistical methods

A systematic testing methodology was established to objectively and reproducibly evaluate the performance of each subsystem and to ensure statistical rigor in the assessment process. All key performance metrics were measured under controlled laboratory conditions with repeated trials. Results are reported as means, standard deviations (SDs), and confidence intervals where applicable.

 Navigation and control performance was verified by repeatedly executing predefined paths within a confined, flat laboratory test field measuring 10 m × 2 m. This scaled setup was designed to isolate and quantify the core path tracking accuracy of the prototype. The accuracy was determined on the basis of the lateral deviations between the actual and target positions of the robot over 60 repeated trials, reported as mean absolute error along with its SD and 95% confidence interval (refer to Table S5). Mechanical actuator performance was verified through physical testing: the positioning accuracy of the lifting mechanism was recorded as the maximum deviation observed over 30 repeated pulse-based positioning tests, while the kinematic consistency of the crank−slider extension mechanism was assessed by comparing the average period measured over 10 consecutive motion cycles with the theoretical period derived from its design parameters. The edge AI pest detection model was evaluated on the basis of a held-out validation set of 120 images using standard object detection metrics, including mAP, precision, recall, and F1 score. Its inference speed was measured as the average latency and FPS over 100 consecutive inferences on the embedded platform. The reliability of the custom communication protocol was assessed via large-scale frame transmission. Baseline data fidelity was calculated from 100,000 consecutively sent frames, the CRC-16 error detection capability was determined by injecting 500 erroneous frames, and the transmission latency was characterized as the mean ± SD from 100 oscilloscope measurements (refer to Table S6).

 The functionality of the integrated system was assessed by verifying the collaborative operation of all subsystems. To confirm real-time closed-loop capability linking perception, decision-making, and actuation, the complete latency from image capture to spray initiation was measured over 50 operational cycles to evaluate consistency. Furthermore, the fundamental operational efficacy of the system was estimated based on laboratory parameters. The area coverage efficiency was theoretically calculated from the simulated moving speed and spray swath, and the operational cost per unit area was projected from the measured average power consumption, local electricity tariffs, and market prices of pesticides (refer to Table S7). This comprehensive testing framework ensures that all reported performance data are traceable and statistically sound and provides a solid basis for establishing the technical feasibility of the proposed integrated system under idealized laboratory conditions. These results serve as baseline performance benchmarks for the proof-of-concept prototype and will inform subsequent field-oriented development.

## Results and discussion

This section presents the component-level verification results for the AI-driven plant protection robot prototype obtained under controlled laboratory conditions. These findings collectively confirm the feasibility of the integrated system architecture for targeted agricultural operations.

### Path planning and motion control performance

Following the methodology described in Sect. 2.6.3, the path tracking accuracy was assessed over 60 repeated trials. The PID controller (Eq. 1) effectively compensated for interwheel speed variations of up to 8% because of motor manufacturing tolerances. The mean path tracking accuracy of the mobile base reached 1.8 cm, with a SD of ± 0.5 cm (Table S5), which is sufficient for interrow navigation in structured environments. The residual error primarily resulted from wheel slippage on the laboratory surface and minor alignment deviations in the grayscale sensor array.

The S-shaped coverage path planner was evaluated through simulation and qualitative comparison analyses. Its operational principle in a structured grid is shown in Fig. 5. Simulation results indicated a high coverage uniformity of 98.2% (Table S7). To assess scalability for larger fields, a comparative analysis was conducted against two classic complete-coverage algorithms, i.e., the Boustrophedon pattern^45^, a simple back-and-forth method, and Spanning Tree Coverage (STC)^46^. As summarized in Table 5, the proposed algorithm achieved a favorable balance: it maintained a near-optimal path length comparable to that of the Boustrophedon algorithm while significantly reducing the number of turns. This reduction is crucial for decreasing the inertial energy consumption and mechanical wear associated with frequent turning maneuvers. In terms of coverage quality, the planner ensured minimal overlap and performed reliably in structured settings. These results demonstrated that by leveraging prior knowledge of crop row geometry, the algorithm effectively streamlines coverage tasks, revealing practical utility for semi-structured agricultural environments.

**Table 5:** Comparison of coverage path planning algorithms.

Algorithm	Primary characteristic	Relative path length	Relative turn count	Coverage quality	Suitable scenario
Proposed S-shaped optimized	Minimizes turns using field structure prior	Near-optimal	Very Low	Minimal overlap	Semi-structured fields
Boustrophedon (Baseline)	Simple, systematic back-and-forth pattern	Short (Baseline)	Very High	No overlap	Unstructured, obstacle-free areas
Spanning Tree Coverage (STC)	Covers decomposed cells via a spanning tree	Moderate to High	Moderate	Can have overlap	Complex, irregularly shaped areas

The combined performance of the precise motion controller and the efficient coverage planner confirms the feasibility of autonomous navigation, providing a foundational capability for subsequent targeted spraying operations.

### Theoretical prediction and consistency analysis of mechanical performance

**Fig. 9:**
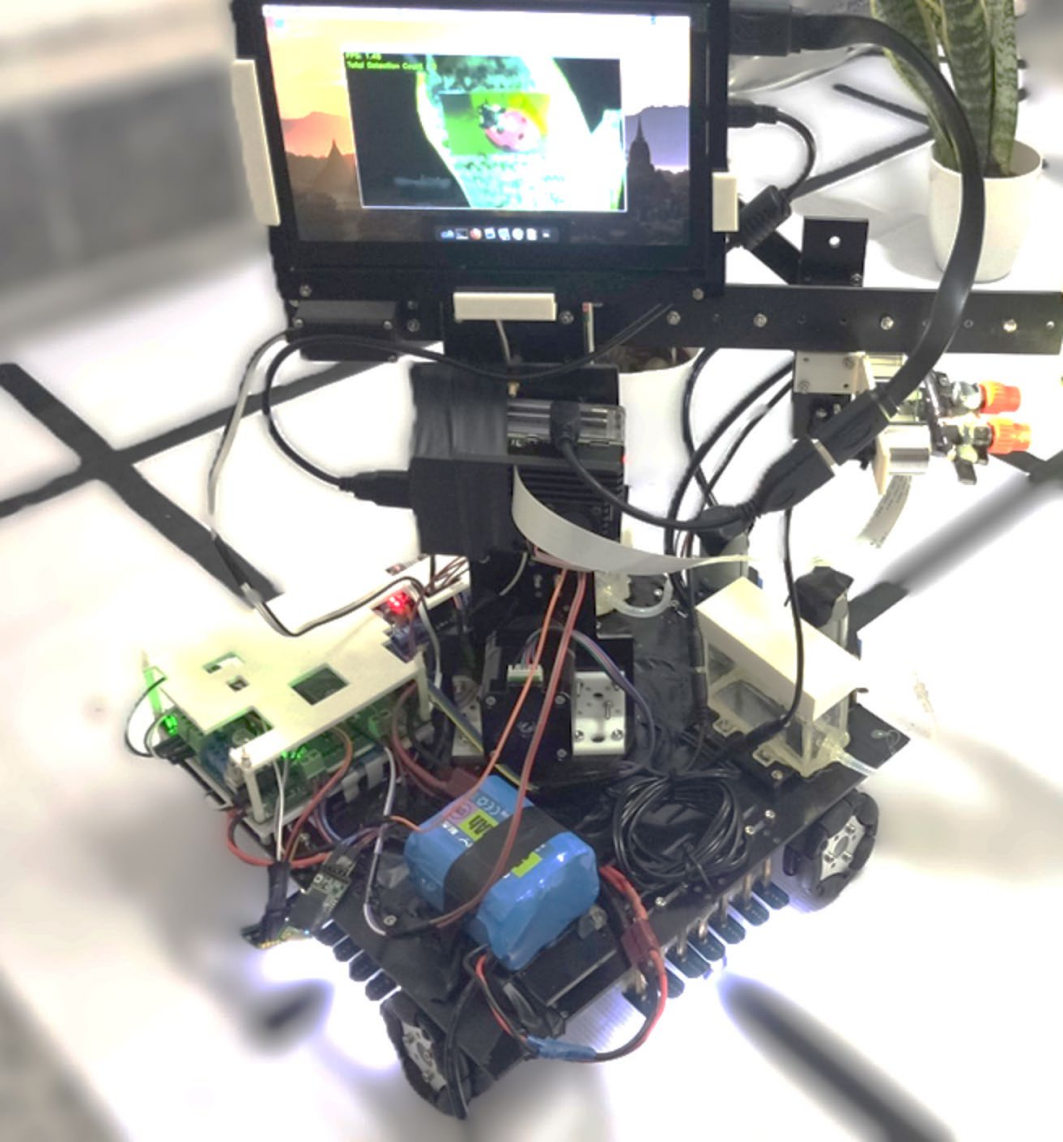
Physical prototype of the integrated plant protection robot.

The physical prototype of the integrated robotic system, which is fabricated according to the designs detailed in Sect. 2, is shown in Fig. 9. The results of initial laboratory tests verified its basic operational capabilities, including holonomic movement, vertical lifting of the robotic arm over its 15–35 cm range, and smooth extension/retraction of the spray head.

Quantitative verification was performed on the basis of key subsystems. The positioning accuracy of the lift mechanism of the robotic arm was assessed by applying calculated pulse sequences (e.g., *N* = 6400 pulses for a 20-mm displacement) and using limit switches for homing. Over 30 repeated trials, the system achieved repeatable positioning accuracy within ± 1 mm, thus confirming the precision of the stepper motor control and belt transmission system.

**Fig. 10:**
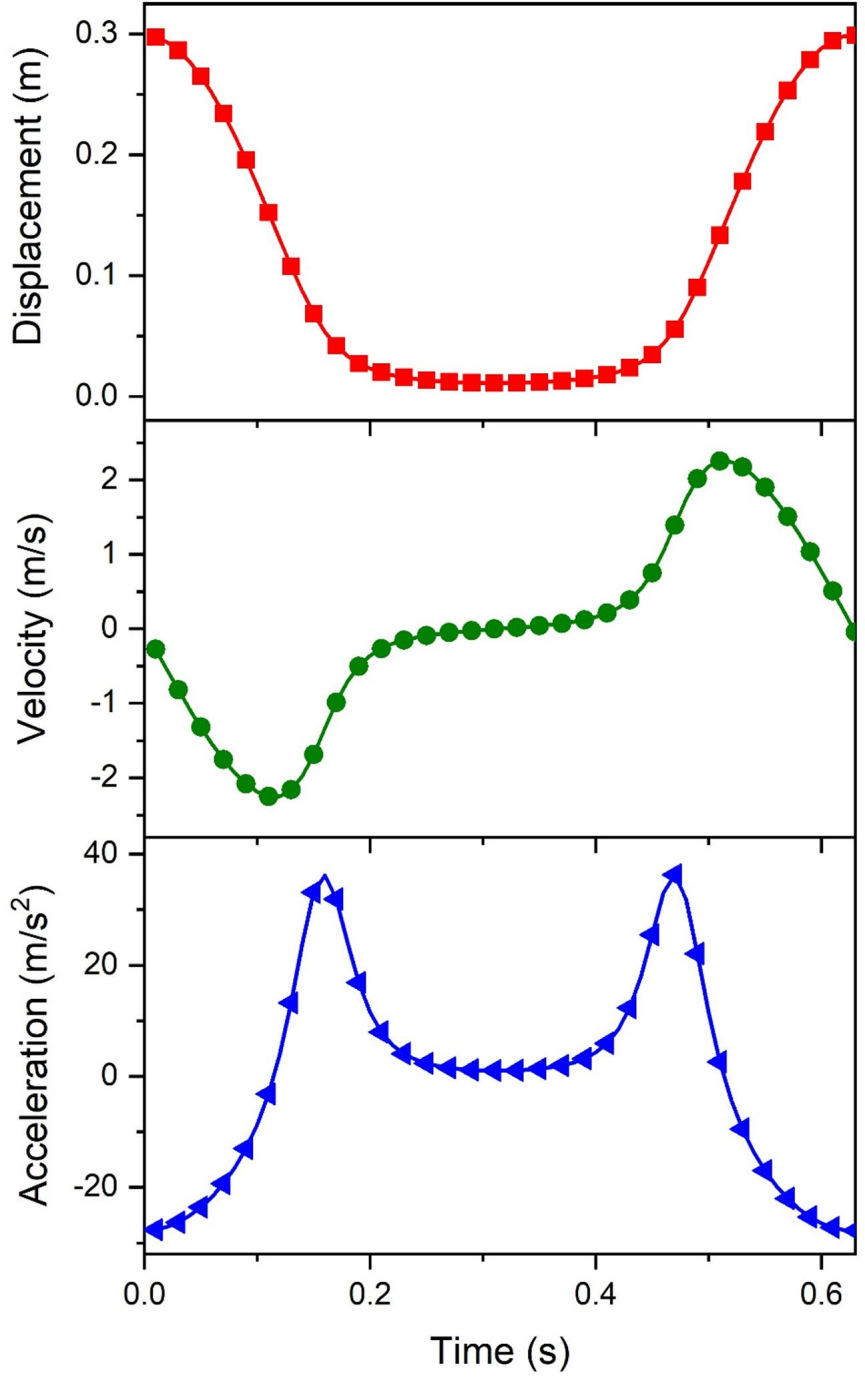
Theoretical kinematic profiles (displacement, velocity, and acceleration) of the crank−slider mechanism over one 0.628-s period.

The theoretical kinematic profiles of the crank−slider extension mechanism, computed from Eqs. 3–5 with the design parameters (*r* = 144 mm, *l* = 155 mm, and *ω* = 10 rad/s), are shown in Fig. 10. The figure shows the displacement, velocity, and acceleration profiles over one complete cycle (theoretical period *T* = 2π/*ω* = 0.628 s). The generated curves exhibit the characteristic periodic variation in nonuniform linear motion inherent to this mechanism.

Close agreement between theory and physical operation was observed: the observed motion was consistent and cyclical, with a cycle time visually matching the theoretical value. Specifically, the displacement curve reaches a maximum at the cycle boundaries (0 and 0.628 s), corresponding to the fully extended position of the slider, and a minimum at the midpoint (~ 0.314 s), corresponding to full retraction. Velocity peaks (negative at ~ 0.11 s and positive at ~ 0.51 s) occur approximately when the crank is perpendicular to the direction of travel of the slider, where the conversion of rotational into linear speed is most efficient. Acceleration peaks emerged at the displacement extremes (0 and 0.628 s) and at ~ 0.16 and ~ 0.47 s, which correspond to instants of motion reversal and to positions where the connecting rod exerts maximum lateral force on the slider, respectively.

The qualitative and quantitative agreement confirms the validity of the kinematic model for functional design. Together with the sub-millimeter positioning accuracy of the lift mechanism, the predictable kinematics of the crank−slider mechanism provide the foundation for the precise nozzle positioning needed in targeted spraying applications.

### Edge AI pest detection performance

The trained YOLOv11l model was evaluated on the basis of an independent validation set of 120 images, following the methodological framework described in Sect. 2.4.1 and 2.6.3. This set included the nine pest classes listed in Table S1, ensuring a representative assessment of generalization.

**Fig. 11:**
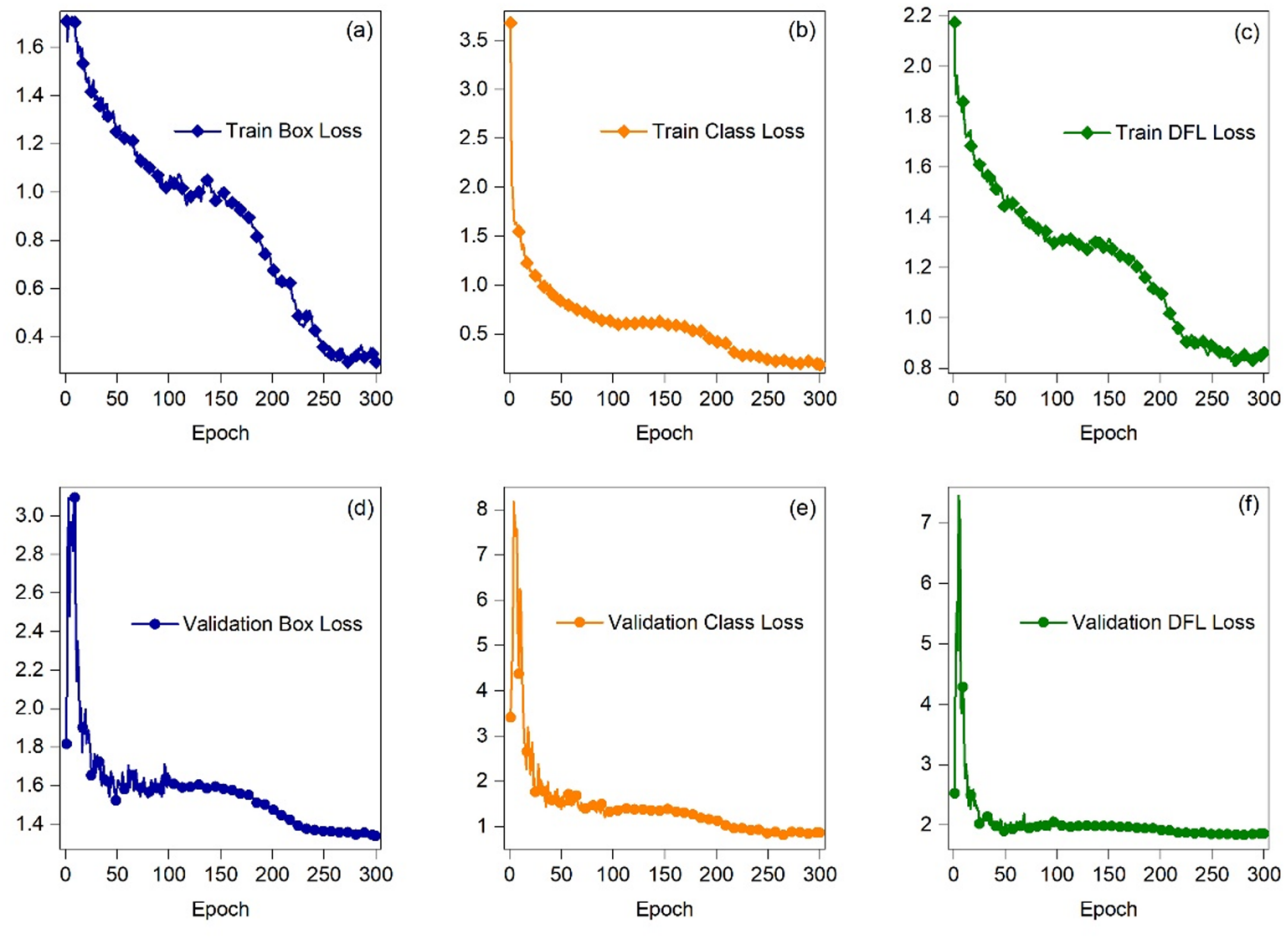
Training and validation loss curves for YOLOv11l. (**a**)−(**c**) show the box, classification, and DFL training losses. (**d**)−(**f**) show the corresponding validation losses. All the curves converge after 300 epochs.

The model demonstrated high efficacy in agricultural pest detection, striking an optimal balance between accuracy and computational efficiency for edge deployment. Training over 300 epochs resulted in robust convergence. As shown in Fig. 11, all training losses decreased steadily, reaching a plateau after approximately 250 epochs. Validation losses followed a similar trend with greater fluctuation, indicating continued adaptation to unseen data without severe overfitting, which justifies the selected training duration.

**Fig. 12:**
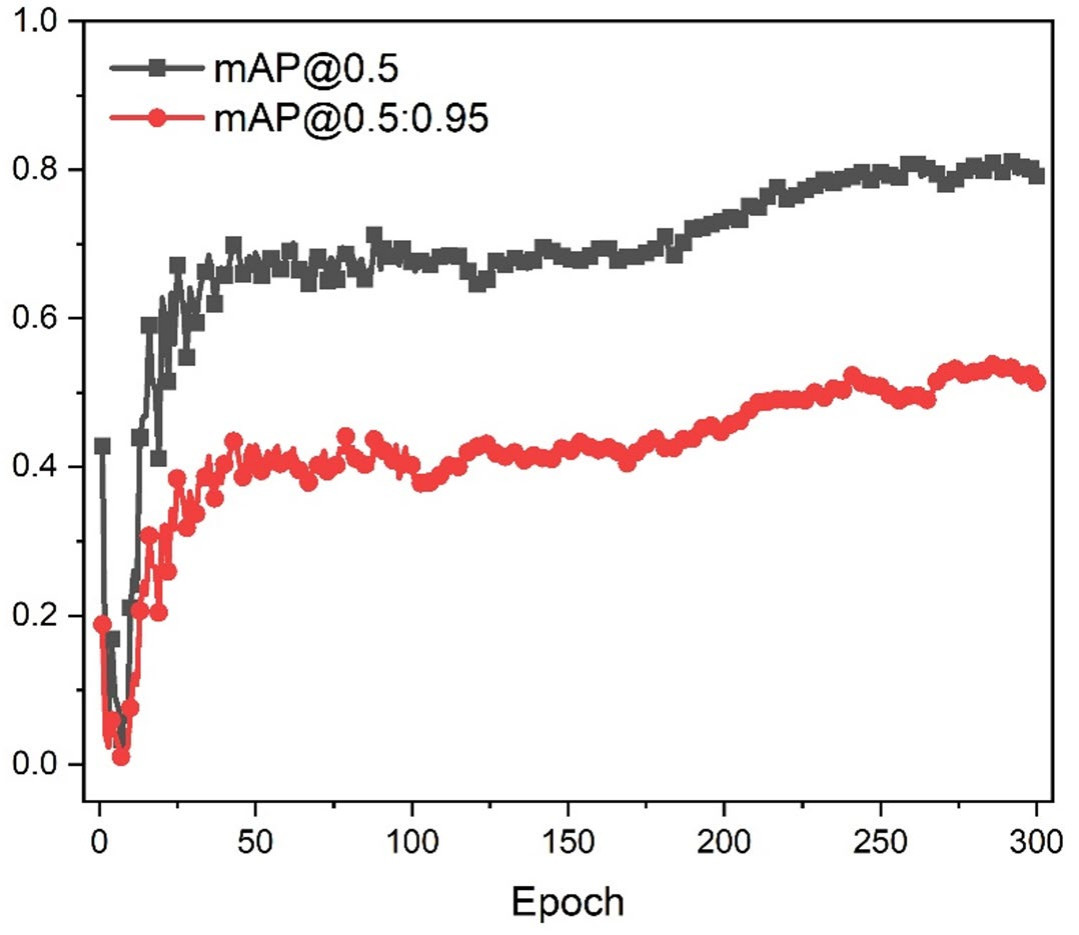
Progression of key mAP metrics for the validation set during training. The curves show the model improvement in both coarse (mAP@0.5) and strict (mAP@0.5:0.95) localization performance over 300 epochs.

Progressive increase in the detection capability is also reflected by the evolution of the mAP during training (Fig. 12). Both mAP@0.5 and the stricter mAP@0.5:0.95 exhibited a consistent upward trend. The most notable gains occurred within the first 50 epochs, after which the metrics continued to improve with reduced volatility, stabilizing at high performance levels.

The final model performance is summarized in Table 6. The model achieved an mAP@0.5 of 0.806 and an mAP@0.5:0.95 of 0.538, indicating high overall detection reliability and competent localization accuracy. The precision (0.821) was higher than the recall rate (0.774), yielding a balanced F1 score of 0.797. This precision-first profile is intentional and desirable for an autonomous sprayer, as it prioritizes the minimization of false-positive activations, thereby reducing unnecessary chemical use and operational cost even at the possible expense of missing pests (especially small or occluded ones), a known challenge in dense foliage. These results confirm the effectiveness of the extensive data augmentation (Table S2) and loss weight configuration (Table S4) outlined in the methodology.

**Table 6:** Evaluation of the performance of the YOLOv11l model for pest detection.

Model	Precision	Recall	F1 score	mAP@0.5	mAP@0.5:0.95
YOLOv11l	0.821	0.774	0.797	0.806	0.538

For deployment, the optimized model was executed on a Raspberry Pi 4B with an inference latency of 35.7 ms per frame (~ 28 FPS). This speed not only represents a standalone metric but also ensures that the perception module operates as a nonblocking component within the larger real-time control loop, whose total latency budget is demonstrated in Sect. 3.5. The achieved accuracy justifies the computational footprint, facilitating reliable, in-field pest monitoring that meets the real-time requirements of the system.

### Communication protocol reliability

**Fig. 13:**
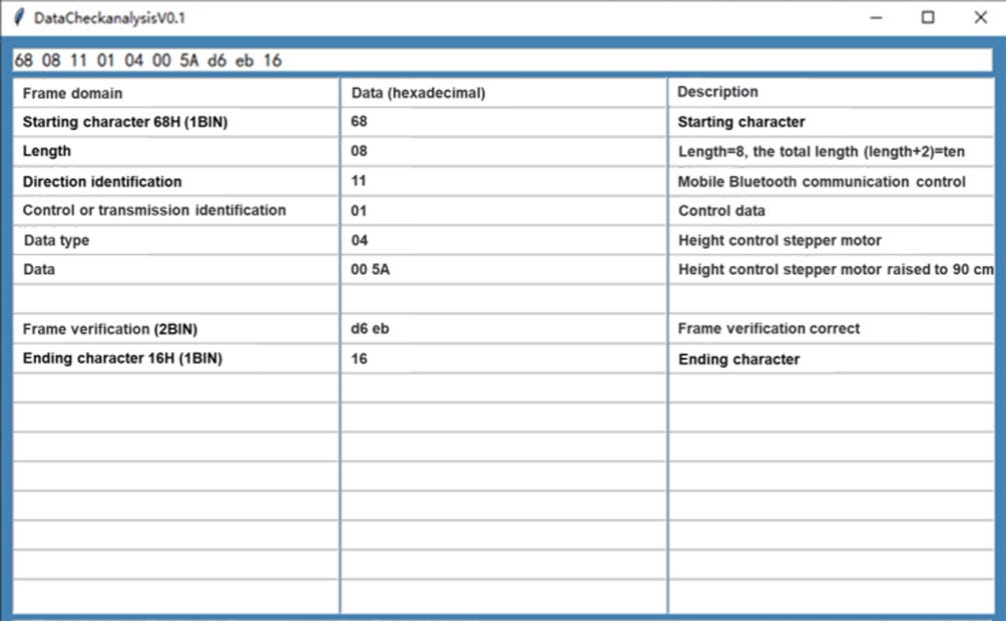
DataCheckAnalysisV0.1 software interface for system monitoring and control.

A series of controlled laboratory tests were conducted to quantitatively assess the performance of the custom communication protocol in terms of reliability, real-time capability, and robustness. The testbed comprised an Arduino Mega 2560 unit (data acquisition terminal), a pair of HC-05 Bluetooth modules forming a 10-m link, and a Raspberry Pi 4B unit (edge processing unit). The entire testing workflow was executed and monitored using our in-house DataCheckAnalysisV0.1 software (Fig. 13), which facilitated frame generation, real-time communication logging, and automated test execution.

Following the procedure described in Sect. 2.6.3, the protocol was subjected to two primary stress tests. First, a continuous stream of 100,000 frames of simulated sensor data (formatted according to Figs. 7 and 8) was transmitted to measure the baseline decoding success rate and latency. Second, to rigorously verify the error-detection capability, 500 deliberately corrupted frames with random bit errors were injected into the data stream (Table S6).

**Fig. 14:**
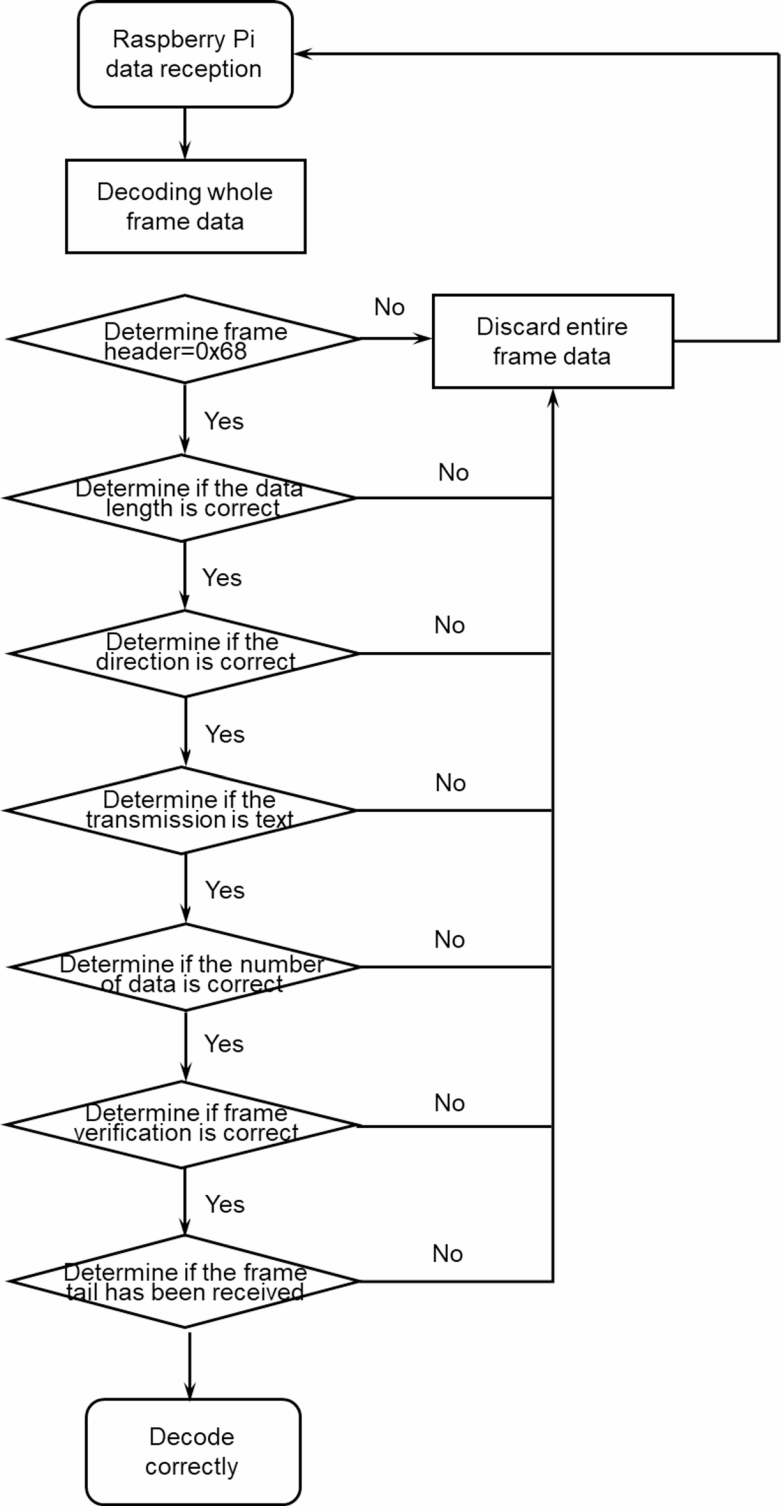
Seven-stage deterministic decoding process of the custom communication protocol, ensuring data integrity through sequential validation checks, including CRC-16 verification.

On the receiver side, each incoming frame was subjected to the rigorous multistage decoding and verification process outlined in Fig. 14. This process ensured robust integrity checking, culminating in CRC-16 checksum verification. Frames failing any check were discarded in real time, thus guaranteeing that only valid data were passed on for processing.

The results, as summarized in Table S6, confirm the high performance of the protocol under laboratory conditions. A frame decoding success rate of 99.91% was achieved, with few failures attributable to sporadic Bluetooth packet collisions, highlighting the value of future testing in representative radio-frequency (RF) environments. The CRC-16 mechanism demonstrated a 100% error detection efficacy against all injected faults. Communication latency was low and consistent (12.3 ± 2.1 ms), satisfying the real-time requirement for critical downlink commands such as an immediate spray trigger. Furthermore, the end-to-end data fidelity exceeded 99.9%, ensuring that subsequent AI-driven decisions were based on accurate inputs.

**Fig. 15:**
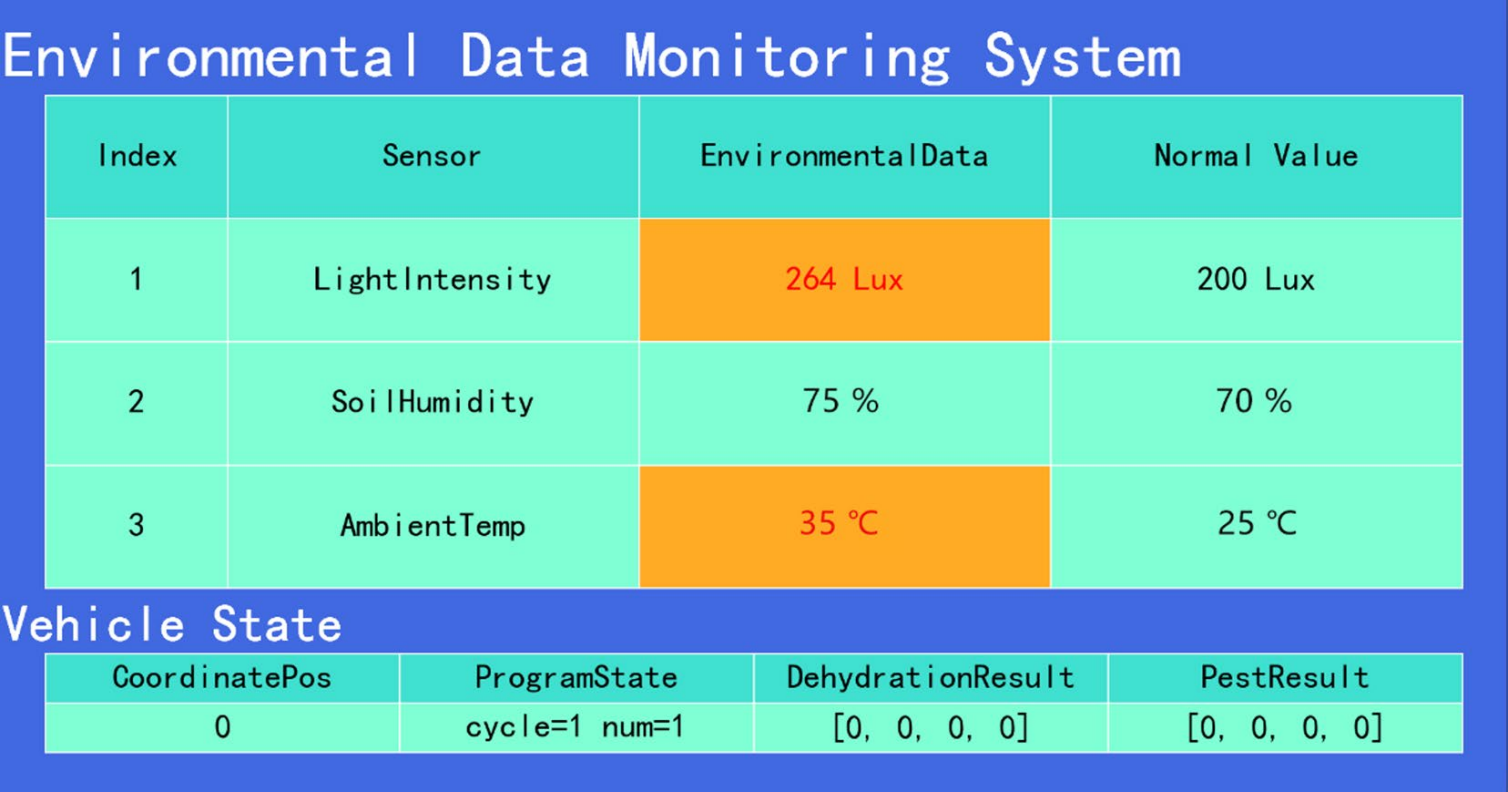
Data analysis results presented on the onboard display of the robot, showing real-time visualization of environmental parameters and derived plant health metrics.

Collectively, these results verify that the lightweight custom protocol meets its core design objectives of high reliability, deterministic low-latency communication, and robust data integrity. The protocol thus forms a dependable data backbone for the integrated system. The practical utility of this link is illustrated by the real-time monitoring interface (Fig. 15), which visualizes decoded parameters and system status, thereby completing the functional chain from data acquisition to operator support.

### System integration and overall efficacy

The ultimate verification of system functionality resides in its capacity to execute closed-loop autonomous operations. This capacity was assessed through a comprehensive end-to-end workflow, encompassing detection, localization, and spraying, designed to evaluate the synergistic performance of all integrated subsystems and the pivotal role of the custom communication protocol.

To quantify integration efficacy, the complete sequence from image capture to spray initiation was executed fifty times under controlled laboratory conditions. The workflow, guided by the protocol-based data architecture depicted in Fig. 6, involved several coordinated stages. Environmental sensor data were transmitted from the distributed node. Concurrently, real-time pest detection was performed by the onboard YOLOv11l model. Upon positive detection, the system generated and transmitted a composite control frame containing target coordinates, arm posture, and spray commands to the master controller. The controller then parsed the instructions, coordinating the navigation and manipulator subsystems to achieve precise nozzle positioning before spray actuation.

This full operational sequence achieved an average end-to-end response time of 190 ± 15 ms, confirming the real-time capability of the system. This performance results directly from the effective interplay of subsystem performance values verified in earlier sections, including the 35.7-ms inference latency of the vision model, the 12.3-ms mean transmission delay of the communication protocol, and rapid mechanical positioning.

**Fig. 16:**
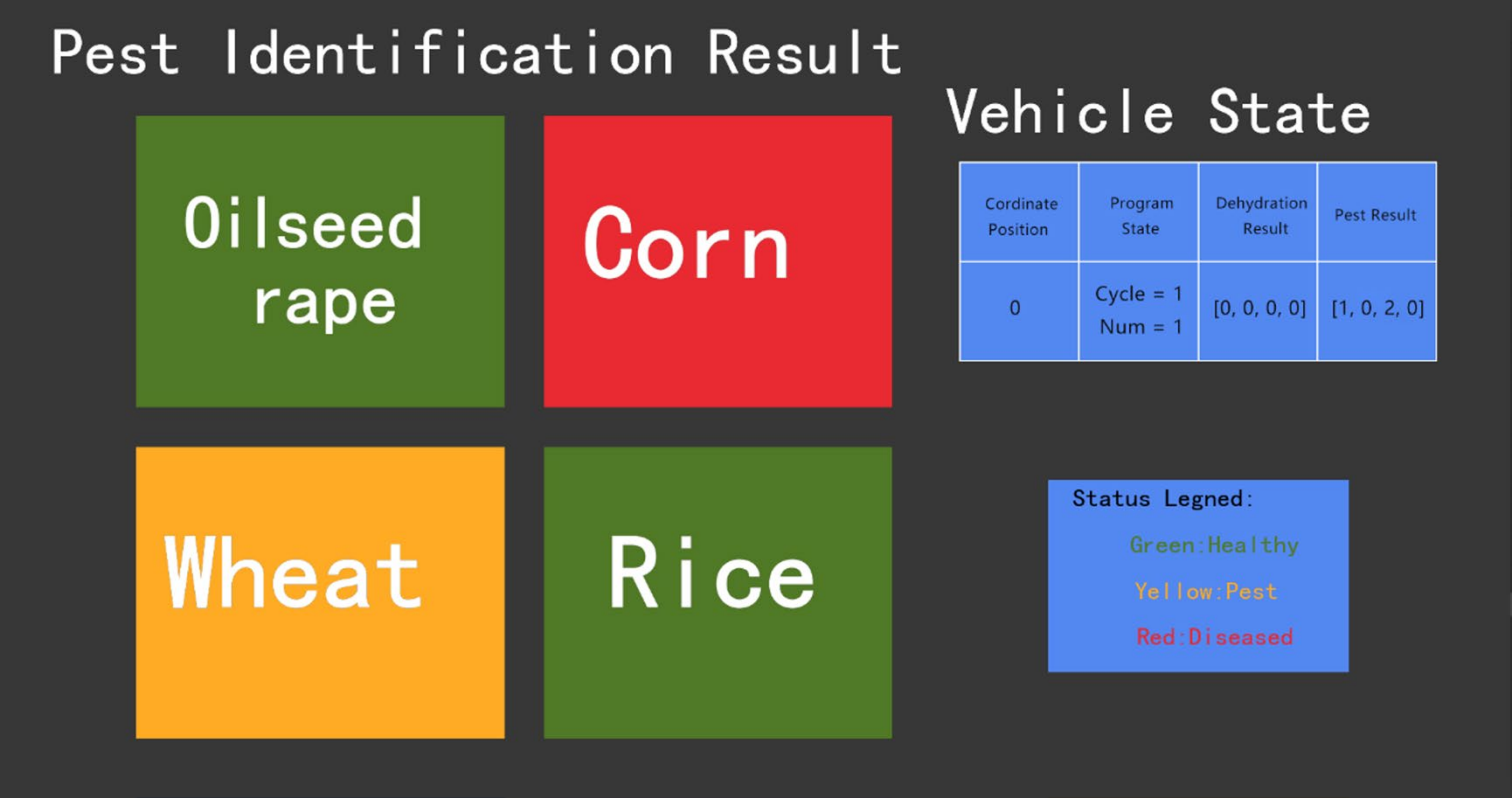
Real-time diagnostic interface of the robot. Detected plant health status is color-coded: green denotes healthy zones, yellow denotes infected regions requiring intervention, and red denotes high-risk areas with severe pest damage.

All critical data conveyed through this integrated pipeline are visualized in real time on the onboard display of the robot, as shown in Fig. 16. The interface provides live sensor readings, annotated pest detection results, and overall system status using an intuitive color-coded scheme for plant health, enabling immediate monitoring.

In summary, the custom protocol serves as the foundational synchronizing layer for deterministic data exchange across heterogeneous hardware components. The successful execution of this closed-loop workflow demonstrates that the individual modules operate as a cohesive autonomous unit. The achieved end-to-end latency, governed primarily by sequential mechanical movements, verifies the practical feasibility of the integrated architecture for real-time precision plant protection in a laboratory setting.

The above results confirm the proposed co-design philosophy. The achieved end-to-end latency of 190 ms and the demonstrated functional reliability under laboratory conditions indicate that prioritizing subsystem synergy and deterministic data flow offers a practical framework for developing effective, low-cost agricultural robotic systems. This approach highlights the value of integrating components to meet system-level objectives over optimizing isolated performance metrics.

### General discussion and limitations

As emphasized in the Introduction, this study represents a proof-of-concept phase in the development of an integrated agricultural robot. The integrated verification presented in this study, while confirming the technical feasibility of the proposed architecture, must be understood within its most significant limitation: all performance evaluations were conducted under controlled laboratory conditions, not in actual agricultural fields. Therefore, the reported metrics reflect the capability of the system under idealized settings rather than its guaranteed operational efficacy in real‑world environments. These results establish baseline performance benchmarks and demonstrate the viability of the proposed co-design approach, providing a necessary foundation for subsequent field-oriented development.

The primary limitation stems from the substantial environmental differences between the laboratory and the field. Critical subsystems face untested challenges outdoors: the precision of the pest detection model has not been evaluated under highly variable natural lighting conditions, complex shadows, or occlusions; the path tracking accuracy was assessed only on flat, uniform surfaces and not on uneven or soft terrain where wheel slippage and navigation errors may occur; and the high reliability of the wireless communication protocol was achieved in a controlled RF environment, rendering it potentially vulnerable to signal attenuation, multipath fading, or interference in dense crop canopies.

These environmental factors directly influence the projected operational and economic metrics. The estimated coverage efficiency and cost per hectare were derived from laboratory-scale simulations under optimal conditions. In practical, large-scale deployment, factors such as adaptive path planning around obstacles, reduced operating speed in rough terrain, battery endurance over full-day cycles, and maintenance logistics significantly influence true economic viability and utility levels. Moreover, the long-term robustness of the system against continuous exposure to dust, moisture, and mechanical vibration inherent to farm environments remains unverified.

Finally, the scope of biological validation is limited. Although the pest detection model performs well for the nine classes included in our dataset, testing against a broader spectrum of pest species, disease stages, and crop varieties encountered across different geographic regions and growing seasons is needed.

These limitations explicitly define the boundaries of the current proof-of-concept study. They do not reduce the value of the integrated co-design approach but rather delineate the key challenges that must be addressed through targeted field validation and engineering refinement, as outlined in the Future Work section.

## Conclusion

### Research summary

This study presented the design and verification of a novel, AI-driven plant protection robot. Its principal contribution lies in a holistic co-design system architecture that demonstrates the feasibility of coupling robust perception, deterministic communication, and precise actuation for real-time agricultural operations. The integrated system successfully unifies real-time pest detection via an optimized YOLOv11l model, multisensor environmental monitoring, precision spraying via a three-axis robotic arm, and reliable data exchange through a custom, lightweight communication protocol. Through component-level testing and system simulation, the platform demonstrated promising performance, with subcentimeter path tracking accuracy (1.8 ± 0.5 cm), a pest detection latency of 35.7 ms on edge hardware, a communication protocol reliability of 99.91%, and a projected operational cost of approximately 1.95 USD per hectare.

It is important to acknowledge that the current verification was conducted under controlled laboratory and simulation conditions. Consequently, the reported metrics reflect the technical feasibility and integrated functionality of the system in idealized settings, not its guaranteed efficacy in unstructured field environments. Key challenges for real-world applications, such as variable lighting, uneven terrain, and wireless interference in agricultural settings, remain to be analyzed.

Despite these limitations, this work provides a concrete and scalable framework that bridges robust hardware design, efficient edge AI processing, and deterministic communication. It establishes a practical foundation for developing cost-effective, autonomous solutions for precision agriculture, with immediate potential for applications in controlled environments (e.g., greenhouses) and as a versatile research platform for advancing resilient farming systems.

### Future work

Building upon this proof-of-concept prototype, future work will focus on enhancing the practical applicability of the system through three key avenues, namely, field validation, algorithmic advancement, and engineered robustness.

- Field validation and environmental robustness: The next step is to conduct comprehensive field trials that aim to evaluate core system functionalities under realistic agricultural conditions. To most effectively close the laboratory−field performance gap, these trials should prioritize three critical aspects. First, communication reliability should be verified for crop canopies by quantifying the packet error rate and effective range under real-world RF interference caused by dense foliage. Second, perception generalization should be assessed by evaluating the YOLOv11l model across different lighting conditions and weather scenarios and against a broader spectrum of naturally occurring pest species. Third, path tracking accuracy should be measured in uneven and soft terrain where wheel slippage is expected, using a real-time kinematic global positioning system (RTK − GPS) as a reference. The outcomes of these prioritized trials will directly inform subsequent algorithmic and hardware refinements.
- Algorithm enhancement and extension: We can enhance the perceptual capabilities of the system by training the detection model on the basis of larger, more diverse datasets that include a wider variety of pest species and disease severity levels. In parallel, we can explore advanced path planning algorithms that can provide dynamic obstacle avoidance and energy-efficient route optimization to increase operational efficiency in complex field layouts.
- System optimization and durability: To ensure long-term operational readiness, engineering efforts should focus on enhancing platform durability and energy autonomy. Key initiatives include hardening hardware enclosures against dust and moisture, exploring supplemental solar charging solutions, and refining mechanical designs for achieving sustained reliability in harsh farm environments.

## Supplementary Information

Below is the link to the electronic supplementary material.

Supplementary Material 1

## Data Availability

Data generated and analyzed during the current study are available from the corresponding author on reasonable request.
