# Supplementary material for: Design and laboratory verification of an AI-driven plant protection robot with a custom communication protocol
Source: Sci Rep. 2026 Apr 18;16:17995. doi: 10.1038/s41598-026-49199-3 (PMC13250158; doi:10.1038/s41598-026-49199-3)
Supplement: Supplementary file 1 — Supplementary Material 1 [file 41598_2026_49199_MOESM1_ESM.doc]

**Supplementary material for**

**Design and Laboratory Verification of an AI-Driven Plant Protection Robot with a Custom Communication Protocol**

Table S1 Pest classes and scientific names.

| **ID** | **Class Name** | **Scientific Name** |
| --- | --- | --- |
| 0 | Mole Cricket | Gryllotalpa |
| 1 | Red Spider | Tetranychus |
| 2 | Flea Beetle | Phyllotreta striolata |
| 3 | Lytta Polita | Lytta polita |
| 4 | Legume Blister Beetle | Epicauta gorhami |
| 5 | Blister Beetle | Meloidae |
| 6 | Oides Decempunctata | Oides decempunctata |
| 7 | Lawana Imitata | Lawana imitata Melichar |
| 8 | Salurnis Marginella | Salurnis marginella Guerr |

**Table S2 Data augmentation configuration.**

| **Augmentation** | **Value** | **Description** |
| --- | --- | --- |
| HSV Hue | 0.015 | Random hue variation for color diversity |
| HSV Saturation | 0.7 | Saturation adjustment for lighting robustness |
| HSV Value | 0.4 | Brightness variation for varied conditions |
| Translation | 0.1 | Random image translation (10% of image size) |
| Scale | 0.5 | Random scaling factor for size variation |
| Flip Left/Right | 0.5 | Horizontal flip probability |
| Mosaic | 1.0 | Mosaic augmentation combining 4 images |
| Erasing | 0.4 | Random erasing for occlusion robustness |
| Auto Augment | randaugment | Automatic augmentation policy |
| Close Mosaic | 10 | Mosaic augmentation disabled in last 10 epochs |

**Table S3 Training hyperparameter configuration for the YOLOv11l model.**

| **Hyperparameter** | **Value** | **Description** |
| --- | --- | --- |
| Epochs | 300 | Number of complete passes through the training dataset |
| Batch Size | 32 | Number of samples processed before model update |
| Image Size | 640 | Input image resolution (pixels) |
| Workers | 8 | Number of CPU threads for data loading |
| Optimizer | Auto | Automatic optimizer selection (AdamW) |
| Initial Learning Rate | 0.01 | Initial step size for weight updates |
| Final Learning Rate | 0.01 | Final learning rate for fine-tuning |
| Momentum | 0.937 | Accelerates gradient descent optimization |
| Weight Decay | 0.0005 | L2 regularization to prevent overfitting |
| Warmup Epochs | 3 | Gradual learning rate increase at start |
| Warmup Momentum | 0.8 | Initial momentum during warmup phase |
| Warmup Bias LR | 0.1 | Bias parameter learning rate during warmup |
| IoU Threshold | 0.7 | Intersection over Union threshold for NMS |
| Max Detections | 300 | Maximum detections per image |

**Table S4 Loss function weight configuration.**

| **Loss Component** | **Weight** | **Description** |
| --- | --- | --- |
| Box Loss | 7.5 | Bounding box regression loss weight |
| Classification Loss | 0.5 | Object classification loss weight |
| DFL Loss | 1.5 | Distribution Focal Loss weight |

**Table S5 Navigation performance during S-shaped path tracking.**

| **Performance Metric** | **Measurement Result** | **Measurement/Analysis Method** |
| --- | --- | --- |
| Path Tracking Accuracy | Mean absolute error: 1.8 cm  Standard deviation: ±0.5 cm  95% confidence interval: ±1.0 cm  (*n* = 60 trials) | Measured on a 10 m straight segment within an S-shaped path. Statistical analysis of repeated trials. |
| Maximum Observed Deviation | 2.2 cm | Worst-case value recorded across all trials. |

**Table S6 Communication protocol reliability.**

| **Performance Metric** | **Measurement Result** | **Test Condition / Analysis** |
| --- | --- | --- |
| Data Fidelity | 99.91% | Ratio of successfully decoded frames to total frames sent (M = 100,000). |
| CRC-16 Error Detection Rate | 100% | Detected all deliberately corrupted frames in an injection test (*n* = 500 frames). |
| Average Transmission Latency | 12.3 ± 2.1 ms  (Mean ± SD) | Measured over a 10-m Bluetooth LE link using an oscilloscope (*n* = 100 trials). |
| Command Execution Accuracy | 100% | All commands were correctly parsed and executed (*n* = 236 test commands). |

**Table S7 Pest detection coverage efficiency and estimated operational cost.**

| **Performance Metric** | **Result / Estimate** | **Test Condition / Calculation Basis** |
| --- | --- | --- |
| Average Coverage Efficiency | 98.7 ± 2.1 m²/h  (Mean ± SD) | Measured in a simulated 10 m × 10 m area using an S-shaped path planning strategy. |
| Coverage Uniformity | 98.2% | Evaluated through a 4-directional spray simulation test (*n* = 10 trials). |
| Path Optimization Effect | 40% reduction in redundant turns | Compared against a conventional back-and-forth path planning method via simulation. |
| Energy Cost Estimate | ~0.52 USD/ha | Calculated based on 60 W average power, local electricity tariff (0.6 RMB/kWh), and coverage efficiency. |
| Pesticide Cost Estimate | ~1.43 USD/ha | Based on the recommended dosage per unit area and average market price of common pesticides. |
| Average Total Operational Cost | ~1.95 ± 0.34 USD/ha | Sum of Energy and Pesticide costs. The variation (±0.34 USD/ha) is derived from the uncertainty in the pesticide cost estimate. |

**Note:** All cost estimates were calculated based on local prices in Chinese Yuan (RMB) and converted to U.S. dollars (USD) using an exchange rate of 1 USD = 6.98 RMB.

**Note on experimental scope:** All performance data presented in this supplementary material and the main manuscript were obtained through controlled laboratory‑based verification. These results establish baseline technical feasibility and are intended to guide subsequent field validation efforts. They do not represent guaranteed performance under real‑world agricultural conditions.
